# Repurposing neuroleptics: clozapine as a novel, adjuvant therapy for melanoma brain metastases

**DOI:** 10.1007/s10585-025-10328-3

**Published:** 2025-01-25

**Authors:** Tobias Wikerholmen, Erlend Moen Taule, Emma Rigg, Birgitte Feginn Berle, Magnus Sættem, Katharina Sarnow, Halala Sdik Saed, Terje Sundstrøm, Frits Thorsen

**Affiliations:** 1 Department of Biomedicine, University of Bergen, Jonas Lies Vei 91, Bergen, 5009 Norway; 2 Department of Neurosurgery, Boston Children’s Hospital, 300 longwood Ave, Boston, MA 02115 USA; 3 Department of Neurosurgery, Haukeland University Hospital, Haukelandsveien 22, Bergen, 5021 Norway; 4 Department of Clinical Medicine, University of Bergen, Jonas Lies Vei 87, Bergen, 5009 Norway; 5 Molecular Imaging Center, Department of Biomedicine, University of Bergen, Jonas Lies Vei 91, Bergen, 5009 Norway

**Keywords:** Melanoma, Clozapine, Cancer, Metastases, Antipsychotics, Brain tumor, Neuroleptic, Drug-repurposing

## Abstract

The blood-brain barrier and the distinct brain immunology provide challenges in translating commonly used chemotherapeutics to treat intracranial tumors. Previous reports suggest anti-tumoral effects of antipsychotics, encouraging investigations into potential treatment effects of neuroleptics on brain metastases. For the first time, the therapeutic potential of the antipsychotic drug clozapine in treating melanoma brain metastases (MBM) was investigated using three human MBM cell lines. Through in vitro cell culture and viability experiments, clozapine displayed potent anti-tumoral effects on MBM cells with an exploitable therapeutic window when compared to normal human astrocytes or rat brain organoids. Further, it was shown that clozapine inhibited migration, proliferation, and colony formation in a dose-dependent manner. Through flow cytometry and proteome screening, we found that clozapine induced apoptosis in MBM cells and potentially altered the tumor immunological environment by upregulating proteins such as macrophage inflammatory protein-1 alpha (MIP-1α) and interleukin-8 (IL-8). In conclusion, clozapine shows significant and selective anti-tumoral effects on MBM cell lines in vitro. Further in vivo experiments are warranted to translate these results into clinical use.

**Graphical abstract:**

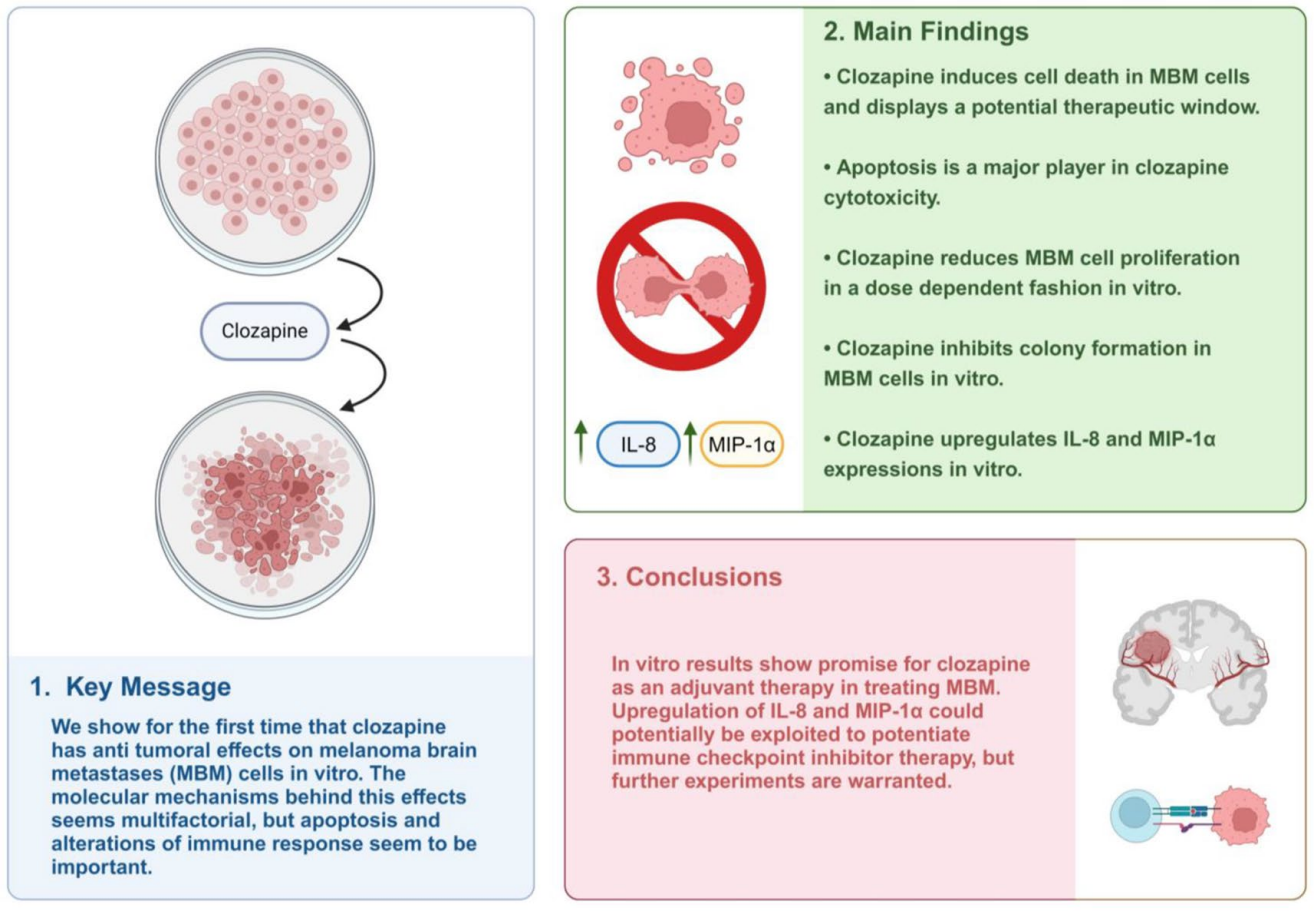

**Supplementary Information:**

The online version contains supplementary material available at 10.1007/s10585-025-10328-3.

## Introduction

Melanoma is a growing healthcare concern, with an incidence of about 324,635 worldwide in 2020. Norway currently has one of the highest incidences of melanoma globally (ranking 5) [1]. Melanoma brain metastases (MBMs) become a major complication for this patient group, as approximately 50% of late-stage melanoma patients develop secondary tumors in their brain, or brain metastases (BM) [2]. The growing incidence and high mortality rates make this of utmost concern to the healthcare system and the scientific community.

Current treatment choices for melanoma brain metastasis (MBM) patients vary with tumor burden and location but commonly consist of surgery followed by combinations of whole-brain radiotherapy, stereotactic radiosurgery, and chemotherapy. Targeted therapies are gradually becoming more personalized for melanoma patients, including BRAF and MEK inhibitors. The recent discovery of immune checkpoint inhibitors has also significantly improved patient outcomes. Unfortunately, many MBM patients do not benefit from these treatments [3, 4].

It has previously been shown that patients with schizophrenia have lower incidences of certain cancer types than the rest of the population. For instance, neuroleptic medication has been associated with reduced risks of cancers in the colon, rectum, and prostate [5]. It has also been shown in epidemiological studies that schizophrenia patients treated with antipsychotic drugs have a lower incidence for glioma [6].

The literature on repurposing neuroleptics in treating BM is still scarce. Preclinically, anti-proliferate effects of neuroleptics on gliomas [7, 8], non-small cell lung cancer (NSCLC) [9], breast cancer BM [10] and MBM [11] have been reported. Studies show that sertindole, fluphenazine, and penfluridol show promising results in treating breast cancer brain metastases in vitro and in vivo [10, 12, 13]. Perhaps most relevant to our current research are three papers showing the cytotoxic effects of fluphenazine and trifluoperazine on MBM cells in vitro [11, 14, 15].

In the current work, we screened a selection of atypical antipsychotics and an atypical antidepressant to increase our understanding of neuroleptic effects on MBM. From our original candidates, clozapine showed significant inhibition of MBM cells in vitro.

## Materials and methods

### Cell lines and cell culture

The Regional Ethical Committee (REC) approved the tissue collection and biobank storage of the tumor biopsies and the development and use of the cell lines (REC Approvals 2013/720 and 2020/65185). Written informed consent was obtained from all patients. The cell lines were authenticated by short tandem repeat (STR) fingerprinting and testing for mycoplasma was done on a regular basis.

The H1, H2, H3, and H10 cell lines were established in our laboratory from MBM patient biopsies. NHA, CHL-1 (CRL-9446), and A375 (CRL-1619) were purchased from American Type Culture Collection (ATCC; Rockville, MA, USA). The Melmet-1 cell line was developed from a subcutaneous metastasis from melanoma and was kindly provided by Ø. Fodstad (University of Oslo, Oslo, Norway).

The H1 patient was operated on for melanoma on the back, and while undergoing radiotherapy of the left axilla, the patient was diagnosed and operated on for brain metastasis. The H3 and H10 patients had only been operated on the thigh and chest for melanomas, respectively. None of the patients had been treated with systemic agents (e.g., chemotherapy, targeted therapy, immunotherapy) or any other treatment directed at their brain metastases. The BRAF mutation status of the H1, H2, H3, and H10 cell lines was determined by massive parallel sequencing of the tumor DNA. The H1, H2 and H10 cell lines are BRAF^V600E^-mutated, whereas the H3 cells are BRAF^L577F^-mutated.

All cells were grown in Dulbecco’s Modified Eagles Medium (DMEM; Sigma-Aldrich Inc., St. Louis, MO, USA), supplemented with 10% heat-inactivated new-born calf serum (Thermo Fischer Scientific, Waltham, MA, USA), 5 µg/mL Plasmocin (Invivogen, Toulouse, France), 2% L-glutamine (BioWhittaker, Verviers, Belgium), penicillin (100 IU/mL) and streptomycin (100 µL/mL) (BioWhittaker). The cells were cultured in a standard tissue incubator at 37 ^0^C, 100% humidity, and 5% CO_2_ and trypsinized once they reached 75% confluency, using 0.25% Trypsin/EDTA (Thermo Fischer Scientific, cat.no 25200056).

### Drug

Clozapine (SelleckChem, Planegg, Germany, cat.no S2495) was dissolved in dimethyl sulfoxide (DMSO; Sigma-Aldrich Inc., cat.no D2438), and stock concentrations of 100 mM were stored at − 80 ^0^C in aliquots of 100 µL.

### Cell viability assay

Two cell viability experiments were carried out to study the effects of treatment on cell viability. First, the cell viability was analyzed in monolayer culture using a Cell Counting Kit 8 (CCK8) cell viability assay (Dojindo Molecular Technologies, Rockville, MD, USA, cat.no CK04). NHA, H1, H2, H3, A375, CHL-1, and Melmet-1 cell lines were seeded at a density of 5 × 10^3^ cells in 100 µL culture medium per well in 96-well plates (Thermo Fischer Scientific, cat.no 167008). The day after, the cells were either left as untreated controls, receiving 100 µL fresh culture medium, or received 100 µL of 2x concentration drug solutions (2, 10, 40, 60, 80, 160, 320 µM) resulting in final drug doses of 1, 5, 10, 20, 30, 40, 80 or 160 µM. The cells were left in drug solutions for a period of 72 h. After treatment, 100 µL of the medium was removed from the 96 wells, and 10 µL of the CCK8 solution was added to each well. The cells were then incubated for 3 h at 37 ^0^C. The absorbance was measured at 450 nm using a plate reader (Thermo Fischer Scientific, Multiscan FC Microplate Photometer) and analyzed with SkanIt software (Thermo Fischer Scientific). In each experiment, 3 wells (*n* = 3) were included for every control and drug concentration, and triplicate experiments were performed. Graphs were made after a blank subtraction, and IC_50_ doses were calculated using GraphPad Prism version 9 (GraphPad Software, Inc., La Jolla, CA, USA). Morphology pictures were taken with a Nikon TE2000 inverted microscope (Nikon Instruments Inc., Melville, NY, USA).

Second, the cells were grown in an anchorage-independent environment using a 3D colony formation assay. H1, H2 and H3 cell lines were seeded in T75 culture flasks (Thermo Fischer Scientific, EasYFlask cat.no 156499) and incubated until a relative confluency of 80%. The cells were then washed with 1xPhosphate Buffered Saline (PBS; Thermo Fischer Scientific, cat.no AM9624), trypsinized using 0.25% Trypsin/EDTA (Thermo Fischer Scientific, cat.no 25200056), and centrifuged (900 rpm for 4 min). The cell number was adjusted to a density of 1.6 × 10^5^ cells/mL and mixed 1:1 with 0.6% low melting point agarose (Sigma-Aldrich Inc.) resulting in a final solution of 8 × 10^4^ cells/mL. Further, 50 µL of the agar-cell solution was then seeded on top of 50 µL of cooled 0.6% Difco noble agar (BD Biosciences, San Jose, CA, USA) in the wells of a 96-well plate (Thermo Fischer Scientific, cat.no 167008), resulting in a total of 100 µL of contents and 4 × 10^3^ cells per well. The low melting point agarose layer with cells was then overlaid with 100 µL of fresh culture medium, and the plate was incubated for 21 days, replacing 100 µL of culture medium every 3 days. At the end of the 21 days, 100 µL culture medium was removed from the wells and 10 µL CCK8 solution (Dojindo Molecular Technologies) was added to each well. The cells were then incubated for 3 h at 37 ^0^C. The absorbance was measured at 450 nm using a plate reader (Thermo Fischer Scientific, Multiscan FC Microplate Photometer) and analyzed with SkanIt software (Thermo Fischer Scientific). In each experiment, 3 wells (*n* = 3) were included for every control and drug concentration, and triplicate experiments were performed. Microscopy images were obtained using a Nikon Eclipse Ti2 microscope (Nikon Instruments Inc.) to assess colony formation. Viability was subsequently analyzed by CCK8 assay as previously described. The analysis was carried out using GraphPad Prism version 9 (GraphPad Software, Inc.).

### Fetal rat brain organoid (FRBO) generation

Pregnant Sprague Dawley rats (Janvier Laboratories, Le Genest-Saint-Isle, France) were sacrificed on the 18th day of gestation under anaesthesia, and fetuses were surgically removed. Under sterile conditions, the fetal brain tissue was removed, combined, and homogenized with scalpel blades. Brain tissue was washed twice with 1 x PBS (Thermo Fischer Scientific, cat.no AM9624) and enzymatically dissociated with StemPro Accutase (Thermo Fischer Scientific, cat.no A1110501) in a 37 ^0^C water bath for 20 min, followed by manual dissociation with a serological pipette. Meninges and other floating debris were removed, and the remaining sedimented tissue was resuspended in medium (DMEM; Sigma-Aldrich Inc.), supplemented with 10% heat-inactivated fetal bovine serum (Thermo Fischer Scientific, cat.no A3840001), 4 mM L-glutamine, 100 U/mL Penicillin/Streptomycin (Thermo Fischer Scientific, prod.code 11659990) and filtered through a 70 μm cell strainer (Thermo Fisher Scientific, cat.no 08-771-2). After two washes in complete medium, single cells were counted, and 2.0 × 10^6^ viable cells were seeded in agar-coated 24-well plates (Thermo Fischer Scientific, cat.no 142485). After three days, aggregated cells were transferred to agar coated T75 flasks (Thermo Fischer Scientific, EasYFlask cat.no 156499). Culture medium changes were performed every 72 h for the first 14 days and every 48 h from day 14 to day 21.

### Live/dead assay

21-day old FRBOs were transferred to 96-low attachment wells and treated with 20 µM, 40 µM, 80 µM, or 120 µM clozapine for 48 h. Untreated organoids were maintained as controls. FRBOs were washed and stained with LIVE/DEAD™ Viability/Cytotoxicity Kit for mammalian cells (Thermo Fisher Scientific, cat.no L3224) according to the manufacturer’s protocol. Briefly, spheroids were incubated for 40 min with 2 µM calcein AM and 4 µM ethidium homodimer-1, transferred to 8-well µ-slides with a glass bottom (Ibidi, Gräfelfing, Germany, cat.no 80826), and imaged. Images were taken using a confocal microscope (Leica TCS SP8 microscope; Leica Microsystem, Germany).

### Proliferation assay

The proliferative capabilities of the cells were studied under constant exposure to drug for 72 h. NHA, H1, H2, and H3 cell lines were seeded at a density of 5 × 10^3^ cells/well in Essen BioScience ImageLock 96-well plates (Essen BioScience Ltd., Hertfordshire, UK, cat. no. 4379) in 200 µL of prewarmed culture medium. After 24 h, the original 200 µL of culture medium was removed and subsequently replaced with drug solutions: 10 µM clozapine, 20 µM clozapine, or 40 µM clozapine (*n* = 6 per treatment group). One treatment group was kept as a control and received only prewarmed culture medium. The culture plate was then placed in an IncuCyte^®^ Live Cell Imaging System (Essen BioScience Ltd.) and imaged every 2 h for 72 h using a 10x objective. The experiment was performed in triplicate (*n* = 3 per experiment per drug concentration). The images were analyzed for confluency using the built-in IncuCyte^®^ Base Analysis Software. The original analysis was then transferred to GraphPad Prism version 9 (Graphpad Software Inc.) for final figure production.

### Migration assay

NHA, H1, H2, and H3 cells were seeded in an ImageLock 96-well plate (Essen BioScience Ltd., cat.no 4379) and left to incubate for 48 h at 37^0^ C. Seeding densities were as follows: H1–3 × 10^4^ cells/well, H2–2.5 × 10^4^ cells/well, H3–2.5 × 10^4^ cells/well. After 48 h, a wound-making tool (Essen BioScience Ltd., IncuCyte wound-maker tool) was used to create a consistent and uniform wound across all the wells. 50 µL of the cell culture medium was pipetted out from the wells, followed by readdition of 50µL of preheated fresh culture medium to remove floating cells. The wells then received 100 µL fresh cell culture medium or with clozapine at 2x concentrations (20 µM, 40 µM, or 80 µM) resulting in final well concentrations of 10 µM, 20 µM, or 40 µM clozapine. Each drug concentration was assigned at least 3 separate wells per experiment. The culture plate was then placed in an IncuCyte^®^ Live-Cell Imaging System (Essen BioScience Ltd.), and imaging was carried out every 2 h for 72 h using the 10 × objective. The wound closure was analyzed using the IncuCyte^®^ Scratch Wound Cell Migration Software Module (Essen BioScience Ltd., cat.no 9600-0012). The experiment was performed in triplicate (*n* = 3 per experiment per drug concentration). The original analysis was then transferred to GraphPad Prism version 9 (Graphpad Software Inc.) for final figure production.

### Clonogenic assay

Clonogenic assays were performed to measure the potential loss of reproductive integrity of MBM cells. The cell lines NHA, H1, H2, H3, and H10 were seeded at a density of 200 cells/well in 2 mL culture medium in 6-well plates (Thermo Fischer Scientific, cat.no 140675) and allowed to attach for 24 h. The cells were then treated with clozapine at 10 µM or 20 µM, for 72 h. Untreated cells were maintained as controls. After 72 h of treatment, the cells were washed with 1 x PBS (Thermo Fischer Scientific, cat.no AM9624), received fresh cell culture medium, and allowed to grow for 12–14 days. After 12–14 days, the culture medium was removed from the wells, and the cell layer was washed with cold 1 x PBS (Thermo Fischer Scientific, cat.no AM9624). The cells were then fixed with prechilled methanol at − 20 ^0^C for 10 min. The methanol was removed, and the plates were left to dry at room temperature. The cells were then stained using 0.5% crystal violet in 25% methanol in water for 10 min. The stain was removed, the cells were washed three times with tap water, and the plate was allowed to dry. The plates were imaged using a Nikon TE2000 inverted microscope (Nikon Instruments Inc., Melville, NY, US). Colonies were counted in ImageJ (National Institutes of Health, Bethesda, MD, USA), and cell clusters of more than 50 cells were registered as surviving colonies. The experiment was performed in triplicate (*n* = 3 per experiment per treatment group). Viability was subsequently analyzed by CCK8 assay as previously described. The analysis was carried out using GraphPad Prism version 9 (GraphPad Software Inc.), and statistics were tested using a two-way ANOVA analysis with multiple comparisons. A p value of less than 0,05 was considered statistically significant. The experiment was performed in triplicate (*n* = 3 per experiment per drug treatment group).

### Apoptosis assay by flow cytometry

Apoptosis was assessed using an AlexaFluor^®^488 Annexin V/dead cell apoptosis kit (Thermo Fischer Scientific, cat.no V13245). For all cell lines (NHA, H1, H2 and H3), 2 × 10^5^ cells were seeded in 5 mL of growth medium in T25 growth flasks (Thermo Fischer Scientific, EasYFlask cat.no 156367). After 24 h of incubation, clozapine was added to the wells at final concentrations of 10 µM, 20 µM, or 40 µM and then incubated for an additional 72 h. Untreated cells were given fresh medium after 24 h, incubated for 72 h, and included as controls. On the day of analysis, the culture medium was transferred to separate sterile 15 mL tubes (Sarstedt, Nümbrecht, Germany, cat.no 62.554.502). The cell monolayers were washed with 500 µL of 1 x PBS (Thermo Fischer Scientific, cat.no AM9624), and the washing solution was transferred into the corresponding tubes. The remaining adherent cells were trypsinized using 0.25% Trypsin/EDTA (Thermo Fischer Scientific, cat.no 25200056), collected, and added to the respective tubes. This was followed by washing and centrifugation at 900 rpm for 4 min. The supernatant was discarded, and 100 µL of an Annexin V binding buffer containing 5 µL Annexin V and 1 µL propidium iodide (Thermo Fischer Scientific, cat.no V13245) was added to each cell pellet and incubated in the dark for 15 min at room temperature (RT). The cells were analyzed using a flow cytometer (BD Bioscience, BD Accuri C6). Fluorescence in the FITC-A and PE-A channels was gated to a two-parameter histogram and analyzed using FloJo software (Tree Star Inc., Ashland, OR, USA). The experiment was repeated three times. The analysis was carried out using GraphPad Prism version 9 (GraphPad Software Inc.), and statistics were tested using ordinary one-way ANOVA tests with multiple comparisons. A p value of less than 0,05 was considered statistically significant. The experiment was performed in triplicate (*n* = 3 per experiment per drug treatment group).

### Human oncology proteome profiler

To elucidate on molecular effects of clozapine, we employed a Human Oncology XL Array Kit (R&D Systems, Minneapolis, MN, USA, cat.no ARY026). Both NHA and H1 cells were assessed. Cells were either left untreated or treated with 30 µM clozapine for 72 h in T75 cell culture flasks (Thermo Fischer Scientific, EasYFlask cat.no 156499). The cells were then counted and lysed at a concentration of 1 × 10^7^ cells/mL in lysis buffer 17 (R&D Systems, Minneapolis, MN, USA, cat.no 895943) containing 10 µg/mL Aprotinin (Sigma-Aldrich Inc., cat.no A6279), Leupeptin and Pepstatin (Tocris, Abingdon, United Kingdom, cat.no 1167/1190). The lysates were centrifuged at 14,000 g for 5 min to remove the cellular debris and quantified using a Direct Detect Spectrometer utilizing the system’s assay-free cards (Sigma-Aldrich Inc., Prod. Nr DDHW00010/DDAC00010). The kit arrays were blocked for 1 h before the buffer was aspirated. To each array, 500 µL cell lysate diluted in a final volume of 1.5 mL of blocking buffer was added and incubated overnight at 4 ^0^C. The arrays were washed three times with a washing buffer. An anti-phospho-tyrosine HRP detection antibody (included in kit) was diluted with the accompanying array buffer and pipetted into the two wells with the arrays. The arrays were incubated for 2 h at RT and washed twice. The accompanying Chemi Reagent Mix was added to develop the protein expression levels. The membranes were imaged with a 1–10 min exposure time using the LAS3000 imaging system (FujiFilm, Saitama, Japan). ImageJ software version 2.0.0 (National Institutes of Health) quantified the relative expressional levels normalized against the reference spots and presented them as a ratio against a negative control. The experiment was performed in triplicate.

### Western blots

To confirm apoptosis after clozapine treatment and verify proteome profiler results, western blotting was used. NHA, H1, H2, H3, and H10 cells were treated with 40 µM for 72 h for apoptosis analysis. To validate proteome profiler results, H1 cells were treated with 30 µM clozapine for 72 h and compared to untreated cells kept in culture medium for 72 h as controls. Cells were lysed using an ice-cold radioimmunoprecipitation assay (RIPA) buffer (Thermo Fisher Scientific, cat.no 09901) supplemented with a cocktail of protease inhibitors and phosphatase inhibitors (Sigma-Aldrich Inc., cat.no 4693124001/04906837001). Cell lysates were centrifuged at 14,000 rpm for 5 min at 4 ^0^C. Total protein was quantified with Direct Detect Spectrometer cards (Sigma-Aldrich Inc., Prod. Nr DDHW00010/DDAC00010). 20 µg of total protein was electrophoresed on 4–12% gradient gels (Thermo Fischer Scientific) for 50 min at 200 V. Samples were transferred to nitrocellulose membrane at 35 V for 90 min. Membranes were blocked for 1 h at RT with 5% skim milk in Tris-buffered saline with 1% Tween (TBS-T) blocking buffer, followed by overnight incubation with primary antibodies at 4 ^0^C: Angiopoietin-Like 4 (cat. no. ab206420, Abcam, BY, LAND), Bcl2 (Abcam, cat.no ab32124), Cleaved PARP-1 (Abcam, cat.no ab32561), Cleaved Caspase-3 (Abcam, cat.no ab32042), CCL3/MIP1α (Abcam, cat.no ab229900), IL-8/CXCL8 (Abcam, cat.no ab289967), HIF1α (BD Biosciences, cat.no 610959), VHL (Cell Signaling, cat.no 68547), β-actin (Abcam, cat.no. ab8224), and GAPDH (Sigma-Aldrich Inc., cat. no. CB1001). Membranes were washed with TBS-T and incubated with secondary antibody for 1 h at RT: HRP-Goat anti-Rabbit IgG (Invitrogen, cat.no 31462), HRP-Goat anti-Mouse IgG (Invitrogen, cat.no 31430).

Similarly, to assess receptor presence in our melanoma cells we performed western blots following the above-mentioned protocol. Primary antibodies used were CHRM3 (1:500, Fisher scientific, cat.no MA538381, lot.no ZH4435985), H4R (1:500, Fisher Scientific, cat.no PA521313, lot.no ZH4435587), D4R (1:500, Fisher Scientific, cat.no PA5104385, lot.no ZH4435227A), CHRNA5 (1:2000, Proteintech.Europe, cat.no 66363-1-Ig, lot.no 10003874), beta-actin (1:4000, Abcam, cat.no ab8227, lot.no 1081220-1). As previously described, the membranes were then washed with TBS-T and incubated for 1 h with secondary antibodies utilizing either our rabbit HRP (1:10000, Beckman Coulter, cat.no IM0831, lot.no 11), or our mouse HRP (1:10000, Cell Signaling, cat.no 7076 S, lot.no 36).

Protein signals were visualized using SuperSignal™ Pico/Femto Chemiluminescent Substrate (Thermo Fisher Scientific) using a LAS-3000 imaging system (Fujifilm). Bands were quantified with ImageJ software (National Institutes of Health) using lane and gel analysis tools. Statistical significance was assessed through two-way standard t-tests in GraphPad Prism version 9 (GraphPad Software, Inc.). Experiments were performed in triplicate, except for VHL which was done in duplicate.

## Results

### Screening of antipsychotics on MBM cells reveals clozapine as a promising drug candidate

**Fig. 1:**
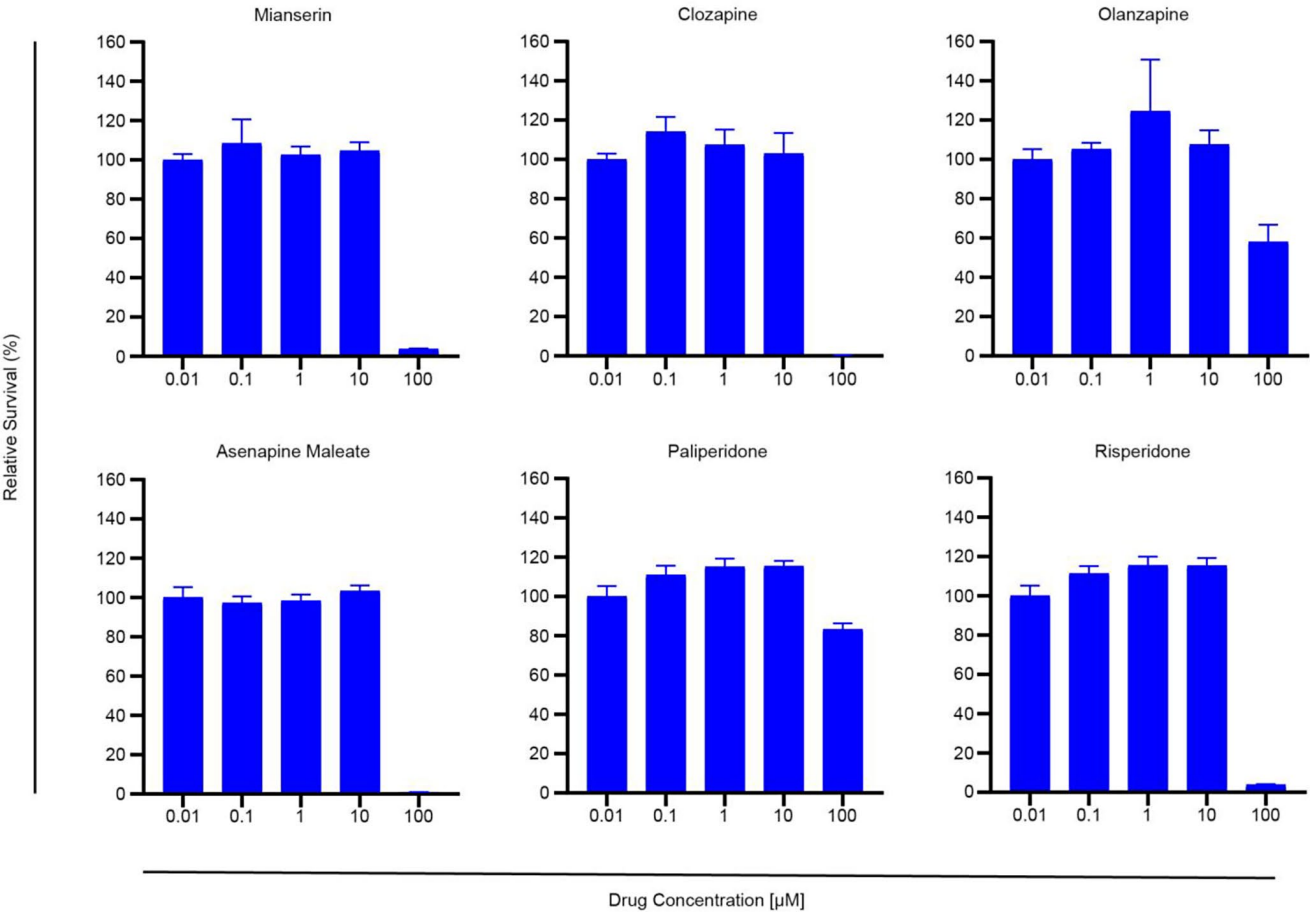
Initial in vitro monolayer drug screening of six drug candidates, at concentrations of 0.01 µM, 0.1 µM, 1 µM, 10 µM, and 100 µM

First, we screened six neuroleptics using the H1 MBM cell line (mianserin, clozapine, olanzapine, asenapine maleate, paliperidone and risperidone). Apparent differences in the cytotoxicity of these antipsychotics were found. Mianserin, clozapine, and asenapine maleate exhibited a markable effect at concentrations of 100 µM, while olanzapine and paliperidone did not display equal cytotoxicity at these levels (Fig. 1). Risperidone also seemed to have a noticeable cytotoxicity on the H1 cell line. However, experiments showed that the apparent cytotoxicity of risperidone was likely due to low DMSO solubility and thus high concentrations of DMSO in the wells at the highest drug concentrations (Online Resource 1).

**Fig. 2:**
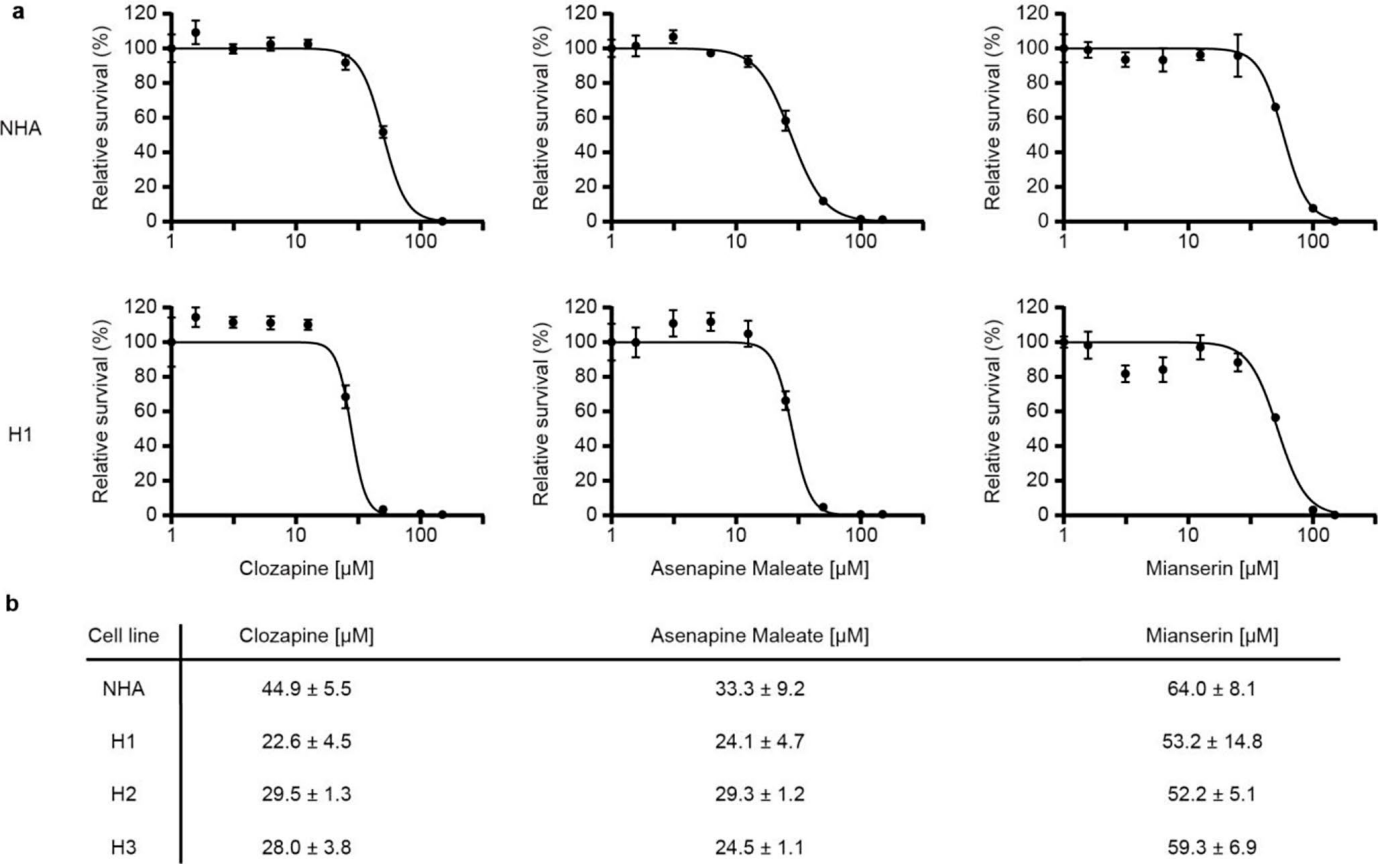
Determination of IC_50_ doses in MBM cell lines in monolayer cultures. (**a**) Representative graphs showing dose-dependent responses of clozapine, asenapine maleate, and mianserin on NHA and H1 MBM cell lines. Variance shown as 1 standard devation (SD). (**b**) Mean IC_50_ doses +/- 1 SD for all cell lines (NHA, H1, H2, and H3) (*n* = 3)

Due to visible differences in viability, our initial screening narrowed the candidates down to mianserin, clozapine, and asenapine maleate. These were therefore selected for further determination of IC_50_ doses on normal human astrocytes (NHA), and the MBM cell lines H1, H2, and H3 (Fig. 2a, b). Clozapine displayed the most considerable difference between the dose required for cytotoxic effect when comparing NHA and MBM cells (i.e. a theraputic window), and was thus selected for further in vitro studies. Similar viability experiments were performed in A375, CHL-1, and Melmet-1 cell lines showing comparable IC_50_ doses (Online Resource 2).

**Fig. 3:**
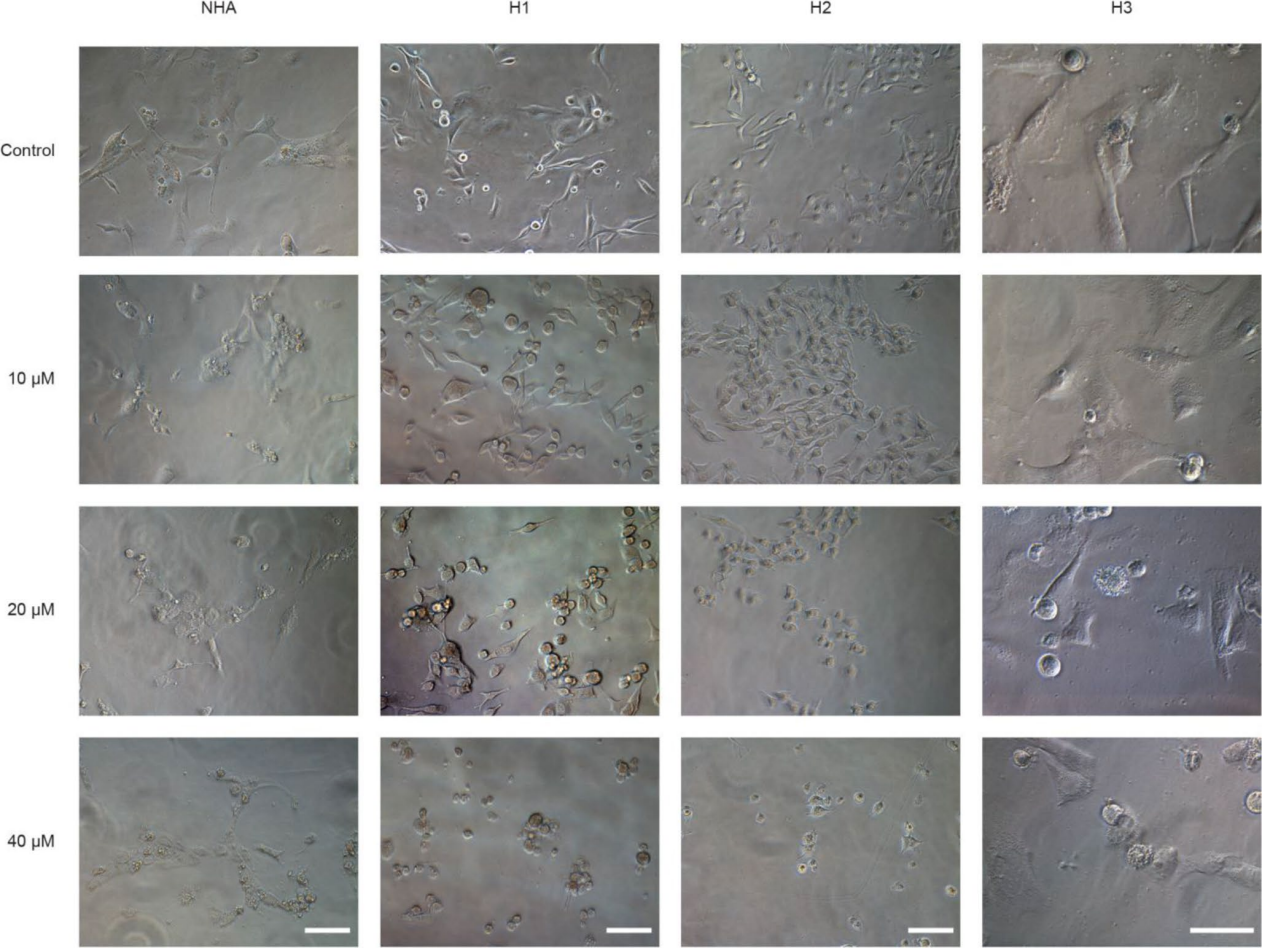
Clozapine alters cell morphology in vitro. Light microscopy images showing NHA, H1, H2 and H3 cells, comparing untreated controls to cells treated with clozapine at doses of 10 µM, 20 µM or 40 µM at 72 h. Scale bar = 200 μm, image columns follow the same scale bar

We then studied morphological changes induced by clozapine after 72 h of treatment with clozapine at doses of 10 µM, 20 µM, or 40 µM. Apparent morphological changes were observed in all cell lines with loss of spindle form, the cells becoming less elongated, smaller, and rounded as the dosage of clozapine increased (Fig. 3).

**Fig. 4:**
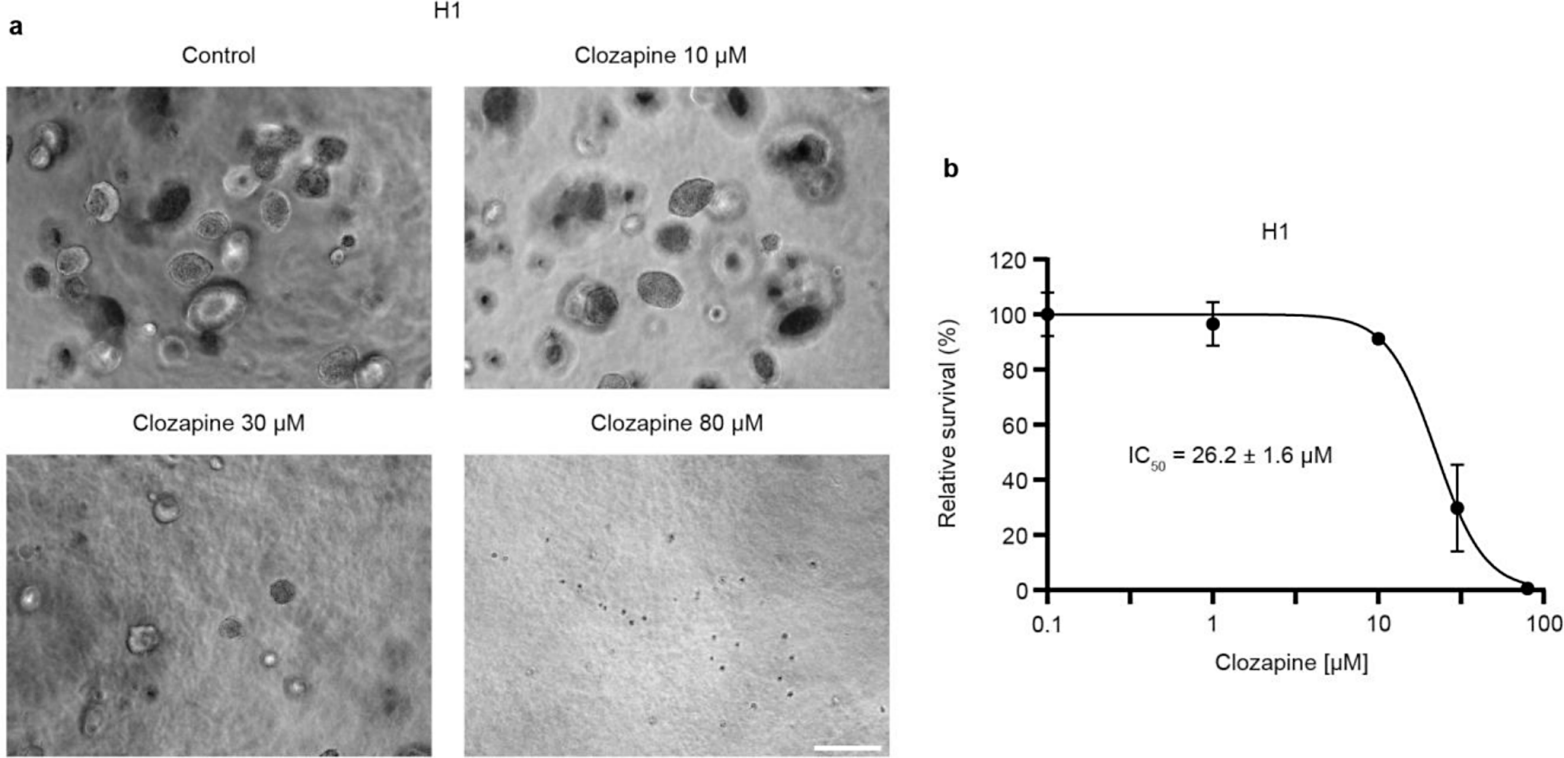
Clozapine effectively inhibits growth in a 3D tumorsphere assay. (**a**) Light microscopy images showing H1 MBM cell colonies suspended in soft agar after 14 days of clozapine treatment as compared to untreated controls. Scale bar = 200 μm. (**b**) Representative figure of IC_50_ doses of H1 cells in an anchorage independent model, graphically represented (*n* = 3)

Next, tumorsphere viability assays were performed to elucidate the effects of clozapine on anchorage-independent growth. H1 and H2 cells showed a dose-dependent decrease in viability after treatment with clozapine, with an estimated IC_50_ of 26.2 µM and 28.5 µM, respectively (Fig. 4 and Online Resource 3). The IC_50_ doses were thus similar to what was found in the monolayer viability studies. NHA and H3 did not grow sufficiently in soft agar and were thus excluded from further studies.

**Fig. 5:**
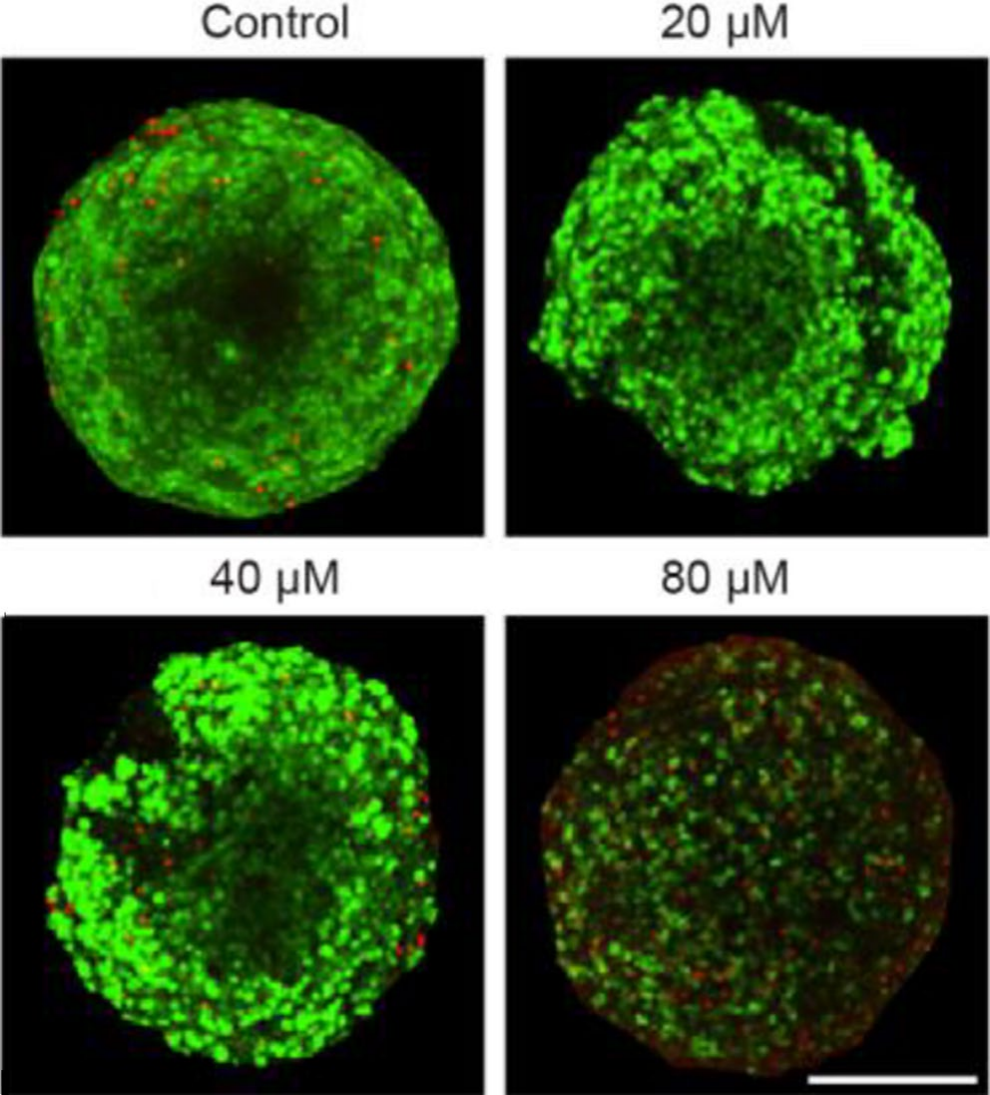
Fetal rat brain organoids (FRBOs) show comparably higher tolerances to clozapine treatment. FRBOs treated with clozapine for 48 h at a concentration of 0 µM (control), 20 µM, 40 µM, or 80 µM. Green color = viable cells, red color = dead cells. Scale bar = 200 μm

To solidify the results on NHA (Fig. 2b), the effects of clozapine on fetal rat brain organoids (FRBOs) were studied. This model system contains all normal brain cells in a 3D structure. The FRBOs were treated with clozapine at doses of 0 µM, 20 µM, 40 µM, or 80 µM for 72 h, stained with a Live/Dead kit, and analyzed qualitatively. Very few dead cells were observed in the organoids at doses up to 40 µM clozapine (green vs. red cells). At 80 µM, we detected a decrease in live (green) cells and an increase in dead (red) cells (Fig. 5).

### Clozapine inhibits MBM cell proliferation in a dose-dependent manner

**Fig. 6:**
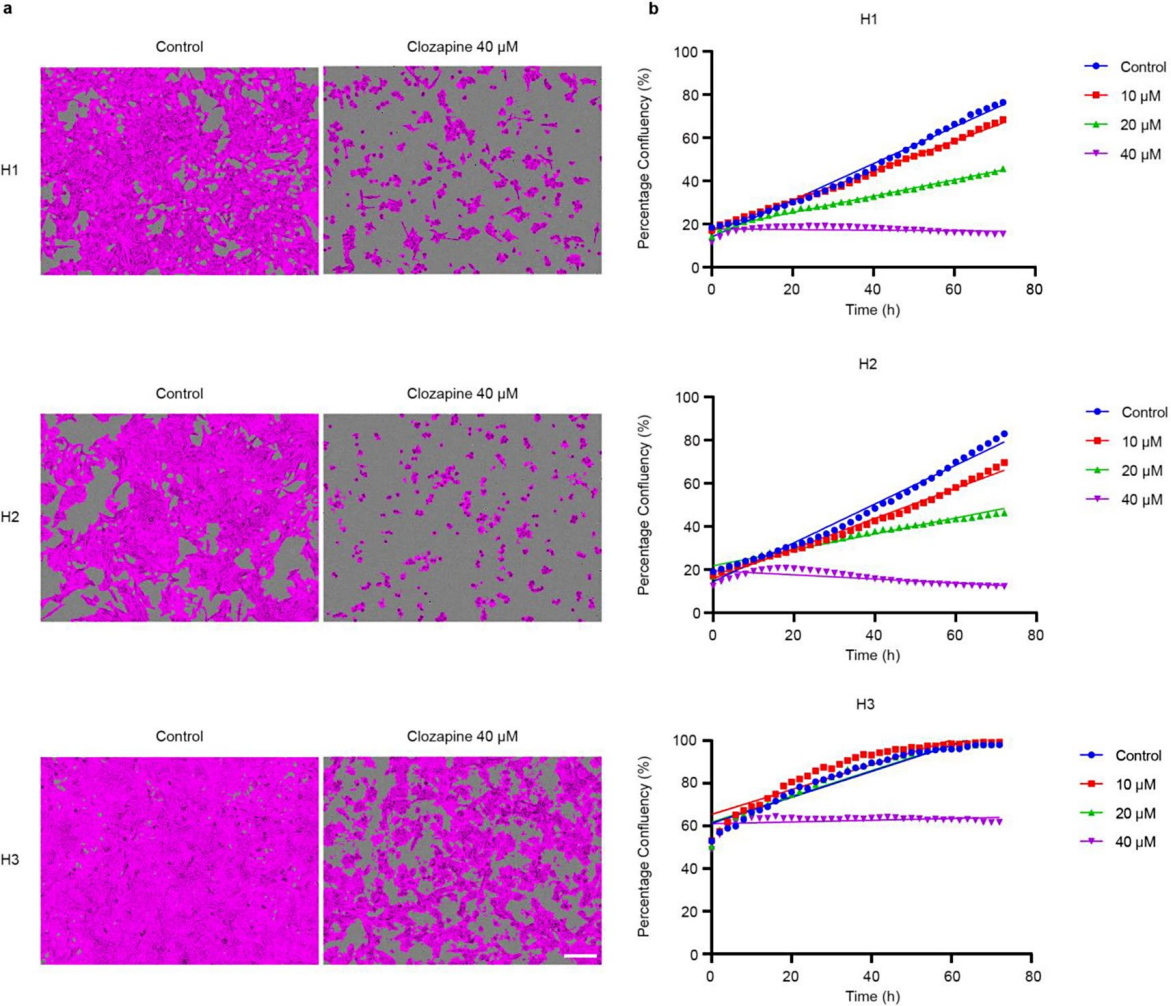
Clozapine inhibits MBM cell proliferation. (**a**) Confluency-masked images from the IncuCyte live cell imaging system show cells as magenta and uncovered areas of the well as gray. The images show wells after 72 h, comparing untreated controls to cells treated with 40 µM clozapine. Scale bar = 200 μm. (**b**) Representative graphs showing well confluency over a time period of 72 h (*n* = 3). For all cell lines 5000 cells were seeded into each well. Due to larger cell size for the H3 cell line, the confluency at time t = 0 was thus higher (60% vs. 20% for the other cell lines)

To study the effects of clozapine on tumor cell proliferation, monolayer proliferation assays were performed using an IncuCyte live cell imaging system. Clozapine effectively inhibited tumor cell proliferation in H1, H2, and H3 cell lines in a dose-dependent manner (Fig. 6a). A dose of 40 µM effectively halted proliferation in all cell lines (Fig. 6b). After treatment with 40 µM clozapine, relative confluencies at 24, 48, and 72 h intervals were 19.0%, 17.4%, and 15.4% for H1, 19.7%, 14.2%, and 12.2% for H2 and 63.1, 63.4, and 61.7% for H3 cell lines respectively.

### MBM cell migration is inhibited by clozapine

**Fig. 7:**
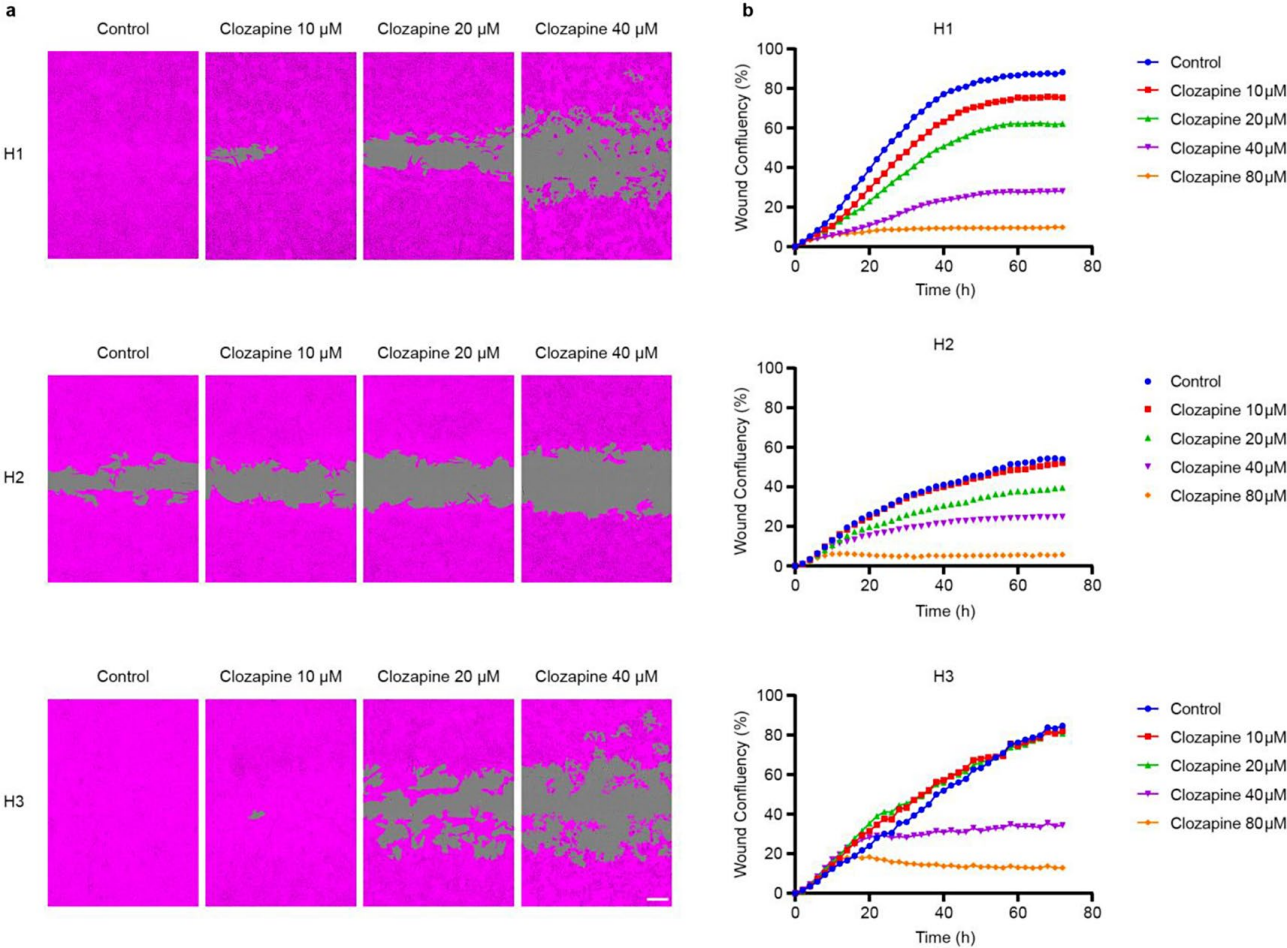
Clozapine reduces MBM cell migration. (**a**) Representative phase contrast images (10 x objective) showing wound closure after 72 h. H1, H2, and H3 cells were treated with clozapine (0 µM, 10 µM, 20 µM or 40 µM). MBM cells are colored magenta, and the scratch wound is colored gray. Scale bar = 200 μm. (**b**) Representative graphs showing wound confluency over 72 h (*n* = 3)

Scratch wound assays were carried out to investigate the effects of clozapine on tumor cell migration. Clozapine inhibited wound closure dose-dependently in all three MBM cell lines (Fig. 7a). At 20 µM, decreased migration was seen in H1 and H2 cell lines. When treated with either 40 µM or 80 µM, migration was effectively impeded in all three MBM cell lines (Fig. 7b). After treatment with 40 µM clozapine, relative wound coverage at 24, 28, and 72 h intervals was 13.2%, 25.9%, and 28.1% for H1, 16.8%, 23.1%, and 24.9% for H2 and 29.3, 33.1, and 34.3% for H3 cell lines respectively.

### Clozapine inhibits MBM cell colony formation

**Fig. 8:**
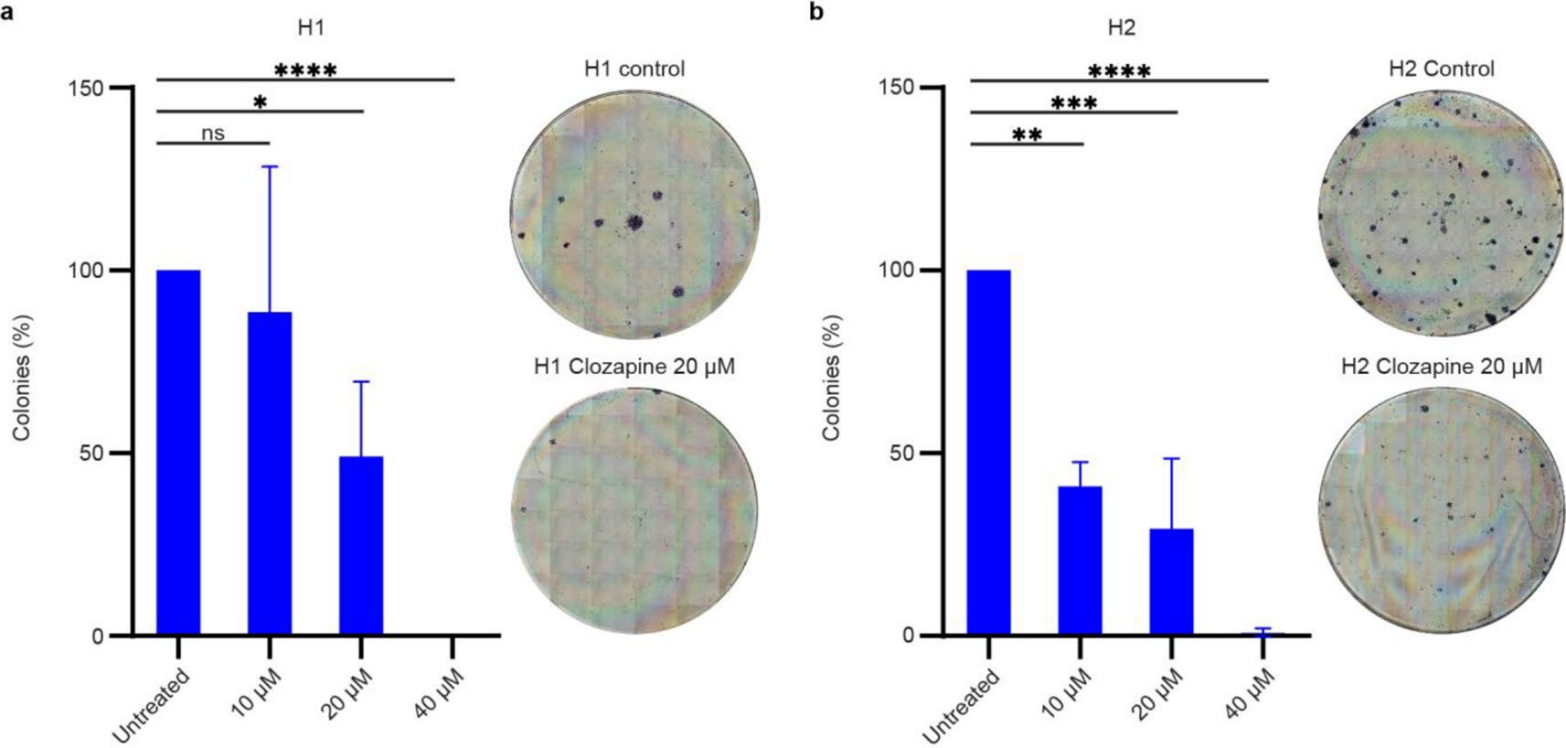
Clozapine reduces MBM cell colony formation in vitro. (**a**) Quantification of colony formation in H1 cells by digital counting of colonies using ImageJ. ns = *p* > 0.05, * = *p* < 0.05, ** = *p* < 0.01, *** = *p* < 0.001, **** = *p* < 0.0001. (*n* = 3). Matching, representative light microscopy images showing full-size wells. Blue stains show cells in rounded colonies on an unstained background. The images show H1 cells treated with clozapine at 20 µM for 72 h as compared to a negative control. (**b**) Quantification and representative light microscopy images of H2 cells following the same schematic as Fig. 8a

To study the potential effects of clozapine on MBM cell colony formation, a 2D colony formation assay was conducted, which is a survival assay based on the ability of a single tumor cell to grow into a colony. Clozapine inhibited colony formation in H1 and H2 cells in a dose-dependent manner (Fig. 8a). Inhibition of colony formation in H1 cells was significant at a dose of 20 µM and 40 µM, while in H2 cells, significant colony formation inhibition was seen at all concentrations of clozapine (Fig. 8b). H3 cells could not form colonies even in control cell cultures and were therefore excluded from this study.

### Clozapine induces apoptosis in MBM cell lines

**Fig. 9:**
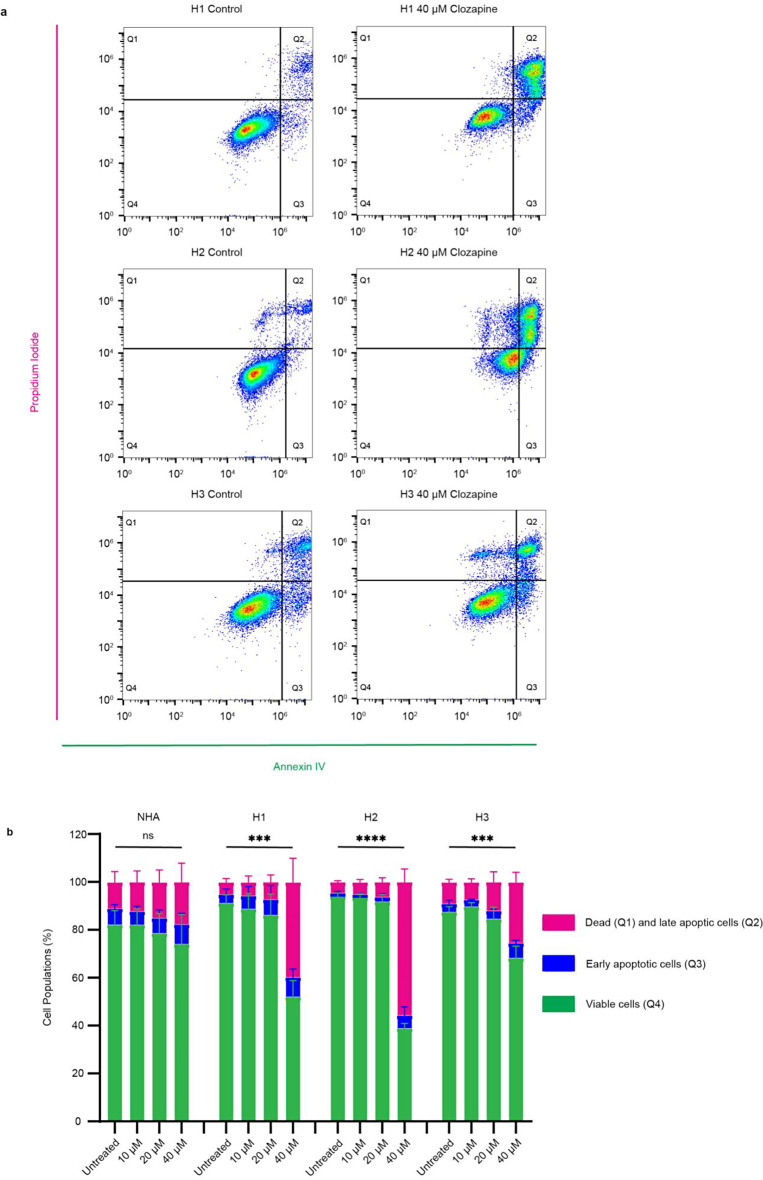
Clozapine induces apoptosis in MBM cells. (**a**) Representative flow cytometry plots showing Annexin V and PI stains of H1, H2, and H3 cells treated with clozapine at 40 µM for 72 h compared to untreated controls. Quartiles are as follows: Q4 – viable cells, Q3 – early apoptotic cells, Q2 – late apoptotic cells, and Q1 - necrotic cells. (**b**) Quantification of flow cytometry results. Q1 and Q2 are combined into a common group representing dead cells and compared across the different drug concentrations. ns = *p* > 0.05, *** = *p* < 0.001, **** = *p* < 0.0001 (*n* = 3). Gating and excerpts from all cell lines including NHA are presented in Online Resource 4

An apoptosis assay was first performed to start elucidating the molecular mechanisms behind the observed inhibitory effects of clozapine. NHA, H1, H2, and H3 cells were treated with 0 µM, 10 µM, 20 µM or 40 µM clozapine for 72 h, stained with Annexin V and PI, and analyzed by flow cytometry (Fig. 9a). At 40 µM, clozapine induced late apoptosis in 43.2% of cells in H1, 42.0% in H2, and 16% in H3, compared to 3.35%, 2.54%, and 8.11% in untreated controls, respectively. NHA showed little apoptosis after 72 h with 40 µM clozapine resulting in 4.85% late apoptosis, while the untreated control showed 7.96% late apoptosis (Fig. 9b).

**Fig. 10:**
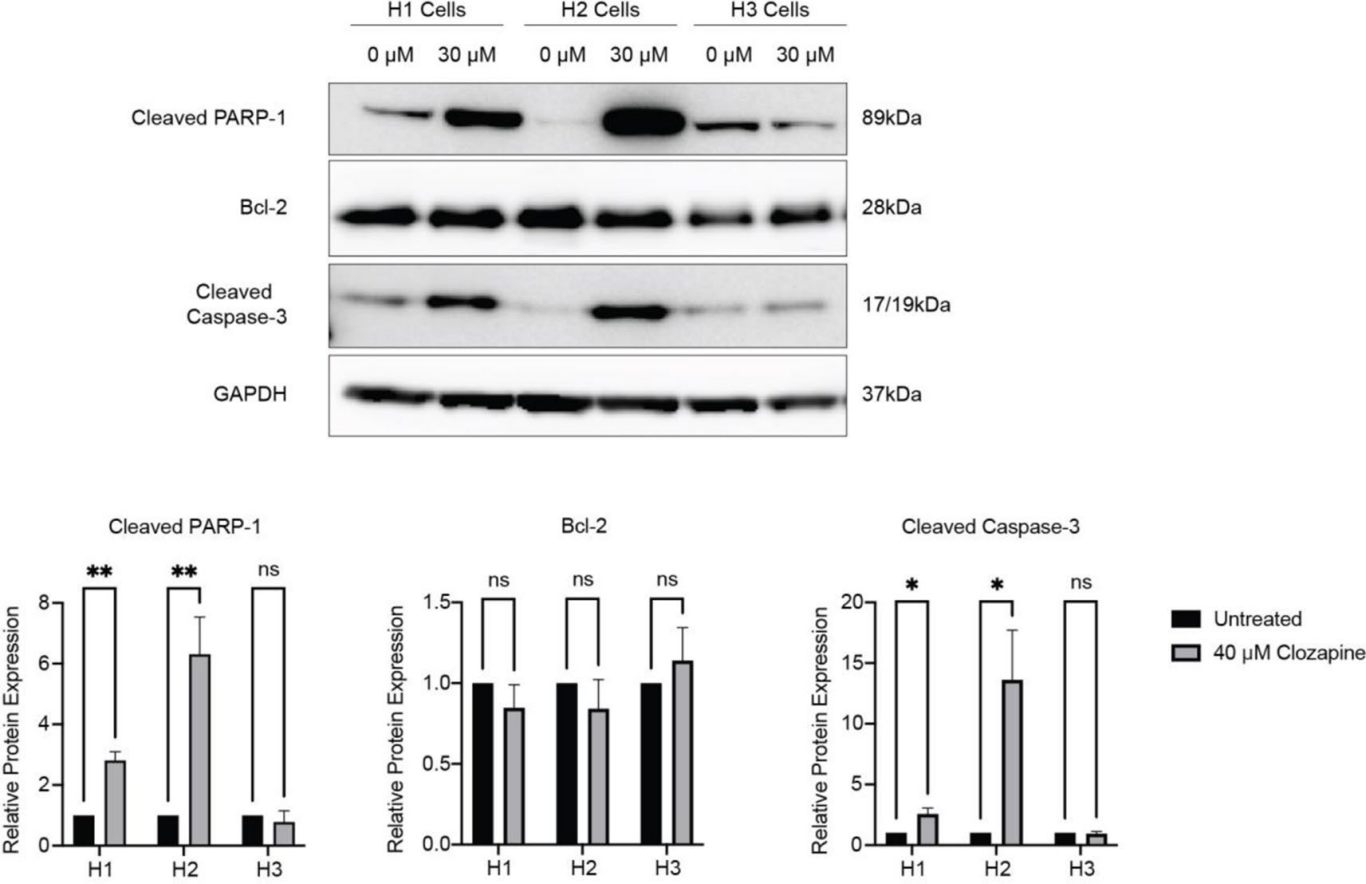
Clozapine treatment induces upregulation of pro-apoptotic proteins. (**a**) Western blots showing cleaved PARP-1, Bcl-2, and cleaved Caspase-3 expression in H1, H2, and H3 MBM cell lines before and after treatment with 40 µM clozapine for 72 h. (**b**) Quantification of changes in protein expression levels before and after treatment with clozapine as measured by pixel density and normalized to glyseraldehyde-3-phosphate dehydrogenase (GAPDH) (*n* = 3). ns = *p* > 0.05, * *p* < 0.05, ** *p* < 0.01. Full-length blots can be found in Online Resource 5

To verify the flow cytometry results, western blots were performed. After treatment with 30 µM clozapine for 72 h, the pro-apoptotic proteins cleaved poly(ADP-ribose) polymerase (PARP-1) and cleaved Caspase-3 were significantly upregulated in both H1 and H2 cell lines (Fig. 10, Online Resource 4). The anti-apoptotic protein B-cell lymphoma 2 (Bcl-2) was not significantly altered after treatment. The H3 cell line showed no significant changes in expression levels of these three proteins after treatment.

### Clozapine targets a wide range of downstream effectors in MBM cells

**Fig. 11:**
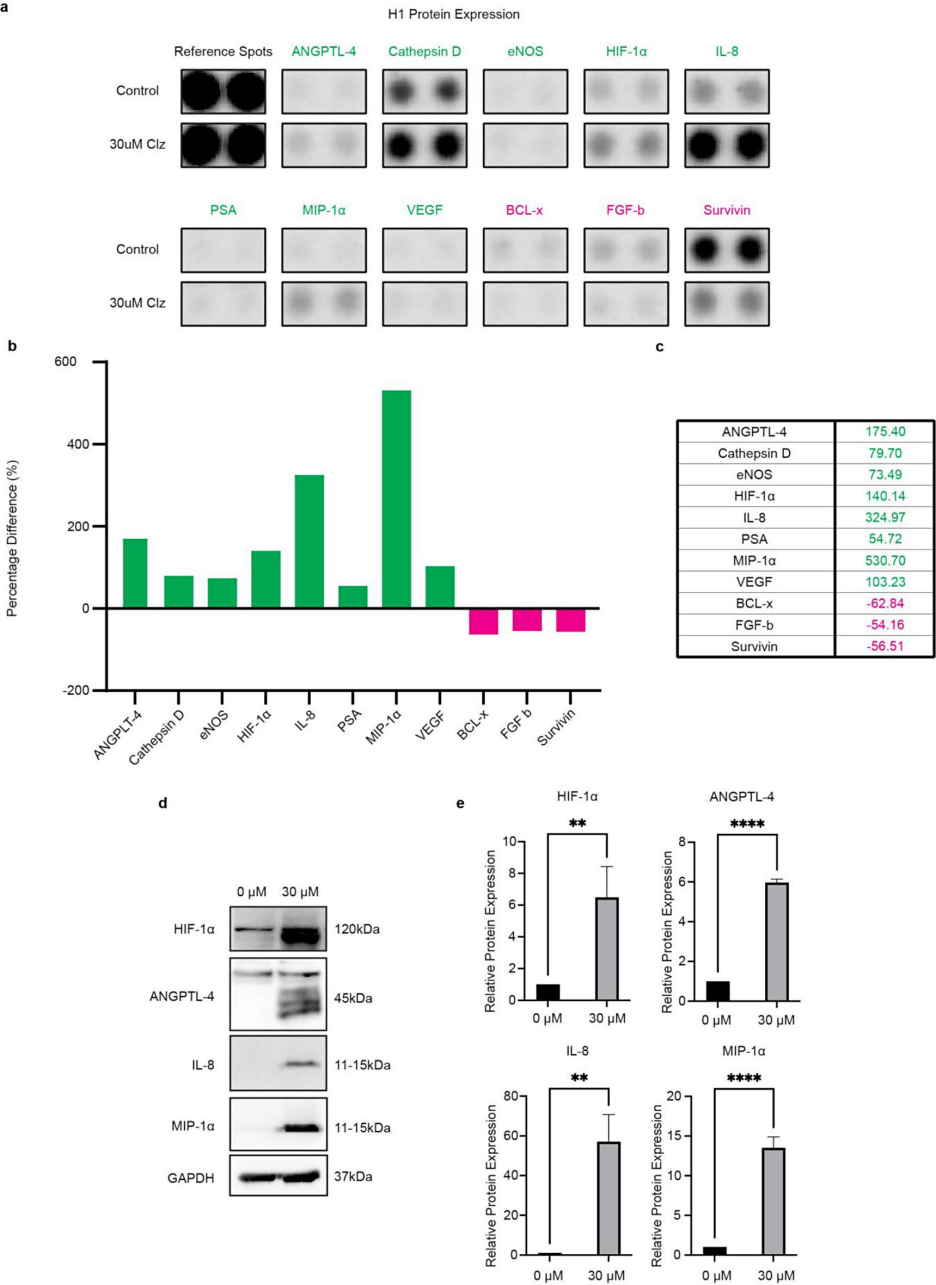
Alterations in cell protein expression levels upon treatment with clozapine. Only proteins with 50% or more in altered expression were selected for presentation. (**a**) Raw blots from the protein profiler. Two blots per sample, comparing H1 cells as untreated controls to 72 h of clozapine treatment at 30 µM. (**b**) A graphical representation of the protein profiler results showing percentage differences in pixel density comparing H1 cells treated with clozapine for 72 h to untreated controls. (**c**) Table showing proteins that were up- or down-regulated by more than 50%; green values are upregulated, and magenta values are downregulated. (**d**) Western blot analysis of the four most upregulated proteins from protein profiler. (**e**) ImageJ quantification of western blots normalized to GAPDH. ns = *p* > 0.05, ** *p* < 0.01, **** *p* < 0.0001 (*n* = 3)

First, we studied wheter our cells MBM cell lines express surface receptors with a previously described affinity towards clozapine. Our cell lines express receptors for histamine 4 receptor (H4R), muscarinic acetylcholine receptor 3 (CHRM3) and nicotinic acetylcholine receptor 5 (CHRNA5), but not the dopamine 4 receptor (D4R) (Online resource 12 and 13). Then, to expand upon the current knowledge of molecular pathways altered by clozapine, we performed a protein profiler array consisting of 84 different proteins selected for their role in known cancer-related pathways. NHA and H1 cells were treated with 30 µM clozapine for 72 h or left as untreated controls, lysed, and immediately assayed. In H1 cell lines, clozapine treatment altered the expression of several interesting proteins. Macrophage inflammatory protein-1 alpha (MIP-1α), Interleukin-8 (IL-8), Angiopoetin-like 4 (ANGPTL-4), and Hypoxia-inducible factor 1-alpha (HIF-1α) displayed the most significant upregulations, while B-cell lymphoma-extra large (BCL-x), Survivin and Basic fibroblast growth factor (FGF basic) experienced the largest relative reductions (Fig. 11a-c). In NHA cell lines clozapine lead to slight alterations of Cathepsin-D and Dkk-1 expressions (Online Resource 6). Full array blots with labels and gating for NHA and H1 cell lines can be found in Online Resource 7 and Online Resource 8, respectively.

From the protein profiler results, the four most upregulated proteins were selected for verification by western blot; MIP-1α, IL-8, ANGPLT-4, and HIF-1α. Compared to untreated cells, expression levels of MIP-1α increased 14-fold, IL-8 55-fold, ANGPLT-4 6-fold, and HIF-1α 5-fold after treatment with clozapine at 30 µM (Fig. 11d-e).

Von Hippel-Lindau protein (VHL) is known to be involved in the degradation of HIF-1α. Western blots elucidating whether VHL downregulation was involved in clozapine’s HIF-1α upregulation were thus performed. Our results show that VHL was expressed in all cell lines, but there was no significant reduction in VHL expression after exposure to 30 µM clozapine for 72 h (Online Resource 10).

Graphical representations of changes in NHA cells can be found in Online Resource 6. Full blots and labeling technique for NHA and H1 cells are presented in Online Resource 7 and 8, respectively. Full-length blots from the WB analysis are presented in Online Resource 9.

Relevant abbreviations: ANGPTL-4 = angiopoetin-like 4, eNOS = endothelial nitric oxide synthase 3, HIF-1α = hypoxia-inducible factor 1 subunit alpha, IL-8 = interleukin 8, PSA = prostate specific antigen, MIP-1α = macrophage inflammatory protein-1 alpha, VEGF = vascular endothelial growth factor, BCL-x = Bcl-2-like protein 1, FGF-b = Fibroblast growth factor basic.

## Discussion

Inhibitory effects of several antipsychotics on brain metastases have been reported previously [9–15]. Our study is the first, however, to investigate the potential effects of the second-generation antipsychotic clozapine in the treatment of MBMs. The current work may be a step towards finding new adjuvant treatments for a group of patients in desperate need of new therapeutic approaches. Recent advances in for instance immunotherapies or targeted therapeutics have not resulted in sufficient survival benefits for MBM patients with a median overall survival still residing at 6.7–10.8 months [4]. This is likely due to unsatisfying response rates and short-lived responses of targeted inhibitors in BM patients. Large scale multi center studies of melanoma patients with BM show that combinatorial dabrafenib/trametinib treatment leads to intracranial response rates of only 58% and median response duration of 6.5 months in BM patients compared to non-BM patients with a median response duration of 12.9 months [16]. With the rise of immunotherapy, the CheckMate 204 trial, utilizing combinatory immunotherapy strategies, has shown that treatment success with immune checkpoint inhibitors largely depends on whether the patient is asymptomatic or symptomatic at the point of diagnosis. For patients with intracranial disease, a study showed a 6-month progression-free survival of 18.9% for the symptomatic group vs. a 62.6% 6-month progression-free survival in the asymptomatic group [17]. This is even more unfortunate as most patients seem to present with neurological symptoms at the point of diagnosis [18]. As a result, new approaches are sorely needed, and neuroleptics might then be a potential supporting player in treating this patient group. Herein we report for the first time the inhibitory effects of the antipsychotic drug clozapine on MBM in vitro.

We selected a wide range of neuroleptics, including one atypical antidepressant (mianserin) and several second-generation antipsychotics representing different degrees of clinical use. Initial screenings of MBM cells showed mianserin, asenapine maleate, and clozapine to have cytotoxic effects at doses below 100 µM (Fig. 1). This was interesting as mianserin had previously been used to treat depression in cancer patients with seemingly good tolerability in clinical studies [19]. Clozapine has a large number of molecular downstream effectors and is widely used in the clinic to treat treatment-resistant schizophrenia [20]. Clozapine was selected due to its favorable therapeutic window and further monolayer viability assays showed comparable IC_50_ doses to previous findings for in vitro lung cancer models (Fig. 2) [9]. When comparing MBM cell lines to primary human melanoma and melanoma skin metastases cell lines (A375, CHL-1, and Melmet-1) in monolayer experiments we find comparable IC_50_ doses, further underlining clozapine’s efficacy on melanoma cells as a whole (Online Resource 2). 3D in vitro tumor models are generally considered more clinically relevant than those in 2 dimensions [21]. In our experiments, the cytotoxicity of clozapine seemed to be anchorage-independent, as IC_50_ doses in 3D cell cultures showed close similarity to those in monolayer culture assays. Interestingly, studies of targeted inhibitors in MBM report significantly reduced IC_50_ doses in 3D cultures compared to monoculture [22, 23]. However, more extensive studies of common chemotherapeutics suggest that IC_50_ doses should increase significantly in 3D in vitro models compared to 2D [24]. Our findings indicate that the cytotoxicity of clozapine is independent of three-dimensional organization and strengthens the findings of our original IC_50_ estimates from monolayer cultures (Fig. 4). Cell morphology changes were increasingly apparent at higher doses of clozapine and might be a direct result of cytotoxicity but might also suggest changes in cell adhesion (Fig. 3).

The FRBO model system has been developed closely mimic the in vivo cell diversity and normal brain morphology more closely [25]. Our qualitative results showed a dose-dependent clozapine cytotoxicity in FRBOs, with a noticeable increase in cell death starting at 80 µM clozapine. The finding that FRBOs are being more resistant to clozapine cytotoxicity than NHA monoculture (80 µM vs. 44.9 µM), suggest that normal brain tissue may be more resistant to effects of clozapine than initially predicted in monoculture (Fig. 5).

Sustained proliferation is a well-established hallmark of cancer and is a well-described phenomenon in MBM [26–28]. Our findings showed a dose-dependent inhibition of proliferation with clozapine treatment (Fig. 6). This might be due to initiation of cell cycle arrest by the treatment, since it has previously been shown that primary human melanoma cells accumulated in the G0/G1 phase after treatment with clozapine [29]. This is underlined by a dose dependent reduction in colony forming abilities as presented through clonogenic assays (Fig. 8). Further, clozapine seemingly affects the migration capacities of MBM in vitro. The migration capacity of primary malignant melanomas is also a well-known feature, and we showed that clozapine inhibited MBM cell migration in a dose-dependent fashion (Fig. 7) [2]. Taken together, it seems that doses well below the estimated IC_50_ of clozapine may inhibit the proliferation, migration and colony formation of MBM cells, which may suggest a potential decrease in formation of microtumors also in a clinical setting.

To understand more about the molecular mechanisms related to clozapine’s observed cytotoxic effects on MBM cell lines, apoptosis was studied by flow cytometry. Clozapine induced significant apoptosis in all MBM cell lines but not NHA (Fig. 9). Clozapine significantly increased the protein expression levels of cleaved Caspase-3 and cleaved PARP-1 in H1 and H2 cell lines (Fig. 10). Caspase 3 is generally known as a master apoptosis regulator and has been shown to regulate the repopulation of tumor sites after treatment-related apoptosis [30]. Cleavage of PARP-1 plays a role in energy conservation, through sparing ATP and NAD, thought to be required for later stages of apoptosis [31]. Bcl-2 is known to prevent apoptosis by several means. Firstly, by preventing the release of mitochondrial apoptotic factors, such as cytochrome c, into the cytoplasm. Alternatively, by inhibiting the proforms of caspases, Bcl-2 might inhibit apoptosome formation [32]. We show no significant upregulations in Bcl-2 expression, which would have counteracted apoptosis. This underlines the proapoptotic activity of clozapine.

Clozapine interacts with a broad range of receptors [33]. Previous studies have shown clozapine to interact with D4R, H4R, as well as acetylcholine receptor of muscarinic and nicotinic type in the form of the muscarinic CHRM3 and the CHRNA5 [29, 34–41]. We show that these receptors, with the exception of D4R, are present in our MBM cell lines and could be a component of clozapine’s anti-tumoral effects. D4R, being the most commonly targeted receptor for atypical neuroleptics like clozapine, is usually expressed in cortical tissue and the mesolimbic system [42]. Previous publications have shown D4R to be present in murine and human primary melanocytes, and stimulation contributes to melanogenesis [43]. The loss of expression in MBM cell lines could perhaps be explained by malignant transformation, and a resulting downregulation of melanogenesis in favor of proliferation. However, to elucidate yet unknown molecular pathways altered by clozapine, we performed a protein profiler array showing a broad change in expression levels following clozapine treatment. The most altered protein expressions were all upregulated, being HIF-1α, VEGF, Angiopoetin-like 4 (ANGPTL-4), MIP-1α, and IL-8, (Fig. 11).

Western blot experiments (Online Resource 10) indicate that VHL downregulation is not the main mechanism of HIF-1α upregulation following clozapine exposure. Therefore, it is more plausible that clozapine induces a general hypoxia in the MBM cells leading to HIF-1α upregulation. It has previously been well established that HIF-1α increases VEGF expression levels, which in turn promotes angiogenesis [44]. However, the literature on HIF-1α as a regulator of IL-8 is somewhat divided. Still, the majority seem to agree that HIF-1α and IL-8 increase proportionally with one another and lead to anti-tumoral effects and increased survival in vivo [45–47]. Others speculate that the nuclear factor κB (NF-κB) pathway plays a central role in the interplay between HIF-1α and IL-8 and their combined antitumoral effects. This is in itself interesting as MIP-1α and other proteins of the CC-motif family are generally thought to be activated by the NF-κB pathway. However, the expression of MIP-1α in relation to HIF-1α seems to vary widely between cell types [48].

HIF-1α is a marker of tissue hypoxia and plays a vital role in erythropoietin production and regulation of glucose transporters. High tumor levels of HIF-1α have previously been shown to be associated with apoptosis and higher survival rates among lung cancer patients [49]. HIF-1α may further counteract apoptosis by inhibiting cytochrome c release and by activating MEK/ERK [50]. As expected, we found an increase in VEGF expression levels in our proteome profiler alongside the HIF-1α increase. This could suggest that the cellular stress induced by clozapine treatment leads the MBM cells to produce HIF-1α and subsequent VEGF seeking to gain nutrition through neovascularization.

ANGPLT-4 is a serum hormone that regulates lipid metabolism and glucose homeostasis [51]. It has been shown that upregulation of this protein prevents metastasis and invasion by reducing apoptosis in vascular endothelial cells [52, 53]. Others find ANGPTL-4 to have pro-angiogenic effects by promoting vascularization in hypoxic conditions [54]. Interestingly, ANGPTL-4 also plays a role in regulating the function of AKT/PKB, which is an upstream effector of glucose consumption and glycolysis during tumor metabolic reprogramming [55]. The exact role of ANGPTL-4 in MBM development would require further studies.

Chemokines play an important role in the tumor microenvironment, and in our study, MIP-1α was overexpressed in the MBM cells after treatment with clozapine. MIP-1α belongs to the C-C motif family and is secreted in a wide range of immune cells [56]. MIP-1α has been shown to attract dendritic cells to the tumor microenvironment and enhance antitumor immunity. Interestingly, it also seems to play a role in T cell activation, as MIP-1α overexpressed in the tumor microenvironment potentiates the effects of PD-1 inhibitors in mouse models [57].

IL-8 is a small cytokine usually produced by phagocytes and mesenchymal cells when exposed to inflammatory stimuli and this cytokine mediates leukocyte tumor infiltration [58]. Though not commonly found in melanocytes, a significant upregulation is often found in melanoma cells [59]. Reviews have evaluated the impact of IL-8 on melanoma growth and metastasis. The results are conflicting, and there are ongoing discussions of its role as a pro-patient vs. pro-tumor cytokine [60]. The observed changes in immune profile (MIP-1α, IL-8) suggest clozapine as a potentiator in immune therapy in combination with for instance immune checkpoint inhibitors. However, before translating the results into the clinic, further studies of the immunological environment in MBM using suitable in vivo models are needed.

Several side effects are associated with clozapine treatment, the most common ones being, drooling, sedation, constipation, weight gain, and dizziness [61]. Severe adverse effects include leukopenia, neutropenia, and agranulocytosis. However, the frequencies of such side effects are comparable to what is observed after using other chemotherapeutic regimens, such as platinum based and vinca alkaloid-based therapies which induce high levels of leukopenia, nephrotoxic and neurotoxic effects, among others [62, 63]. Further, targeted therapeutics currently in use clinically also carry similar risks of the same low-grade side effects [64]. Thus, we believe that our in vitro data shows clozapine to be a promising candidate for further research on the role of neuroleptics in the development of novel cancer therapeutics. However, further in vivo experiments are warranted to support clinical application.

## Conclusions

We show for the first time that clozapine exhibits dose-dependent inhibition of cell viability, proliferation, migration, and colony formation in human MBM cell lines. Clozapine induces apoptosis by upregulating cleaved Caspase-3, cleaved PARP-1, and downregulating Bcl-2. Simultaneously we show that clozapine alters many known cancer-related proteins, including interesting immune regulatory proteins like MIP-1α and IL-8. These findings might suggest clozapine as a possible potentiator of immune therapy. However, additional in vitro and in vivo studies are warranted to support the translation of clozapine as an adjuvant treatment for patients with MBM.

## Electronic supplementary material

Below is the link to the electronic supplementary material.

Supplementary Material 1

Supplementary Material 2

Supplementary Material 3

Supplementary Material 4

Supplementary Material 5

Supplementary Material 6

Supplementary Material 7

Supplementary Material 8

Supplementary Material 9

Supplementary Material 10

Supplementary Material 11

Supplementary Material 12

Supplementary Material 13

Supplementary Material 14

Supplementary Material 15

## Data Availability

The datasets used and analyzed during the current study will be provided by the corresponding author upon reasonable request.

## Abbreviations

AKT: Protein kinase B
ANGPTL-4: Angiopoetin-like 4
BCL-x: Bcl-2-like protein 1
Bcl-2: B-cell lymphoma 2
BRAF: v-Raf murine sarcoma viral oncogene homolog B1
CCK8: Cell counting kit-8
CCL3: Chemokine (C-C motif) ligand 3
CO2: Carbon dioxide
DMSO: Dimethyl sulfoxide
DNA: Deoxyribonucleic acid
EDTA: Ethylenediamine tetraacetic acid
eNOS: Endothelial nitric oxide synthase 3
FGF-b: Fibroblast growth factor basic
FITC: Fluorescein isothiocyanate
FRBO: Fetal rat brain organoids
GAPDH: Glyceraldehyde 3-phosphate dehydrogenase
HIF1a: Hypoxia-inducible factor 1 subunit alpha
HRP: Horseradish peroxidase
IC_50_: Half maximal inhibitory concentration
IL-8: Interleukin 8
MBM: Melanoma brain metastasis
MEK: Mitogen-activated protein kinase kinase
MIP-1α: Macrophage inflammatory protein
NHA: Normal human astrocytes
OS: Overall survival
PARP-1: Poly (ADP-ribose) polymerase
PI: Propidium iodide
PSA: Prostate specific antigen
REC: The Regional Ethical Committee
RIPA: Radioimmunoprecipitation assay
RT: Room temperature
STAT: Signal transducer and activator of transcription
STR: Short tandem repeat
TBS: Tris-buffered saline
TCA: Tri-cyclic antidepressants
VEGF: Vascular endothelial growth factor
VHL: Von Hippel-Lindau protein
Wnt: Wingles-related integration site
WT: Wild-type
3D: Three dimensional
µM: Micromolar
µm: Micrometers
