# Supplementary figures and images for: Repurposing neuroleptics: clozapine as a novel, adjuvant therapy for melanoma brain metastases
Source: Clin Exp Metastasis. 2025 Jan 25;42(2):12. doi: 10.1007/s10585-025-10328-3 (PMC11761981; doi:10.1007/s10585-025-10328-3)

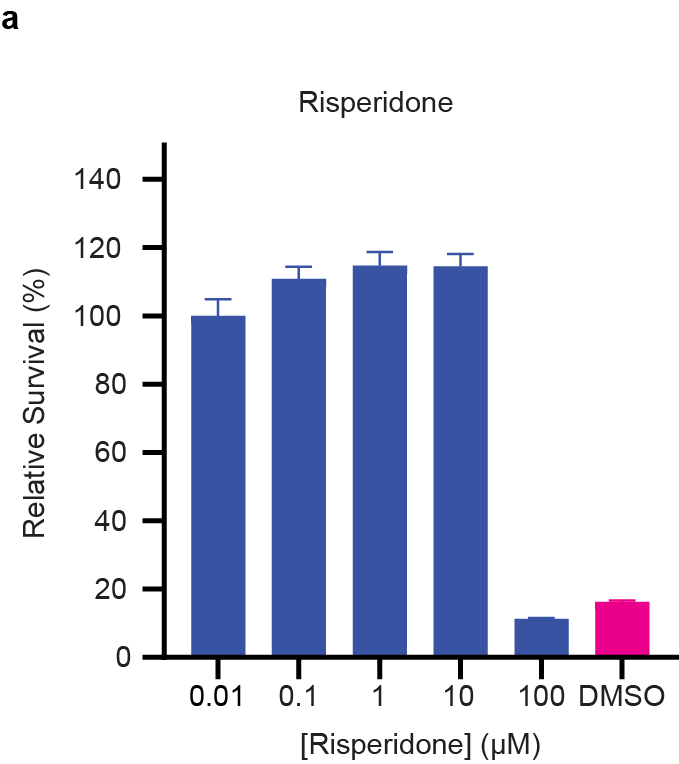

Supplement: Supplementary file 1 — Supplementary Material 1 [file 10585_2025_10328_MOESM1_ESM.jpg]

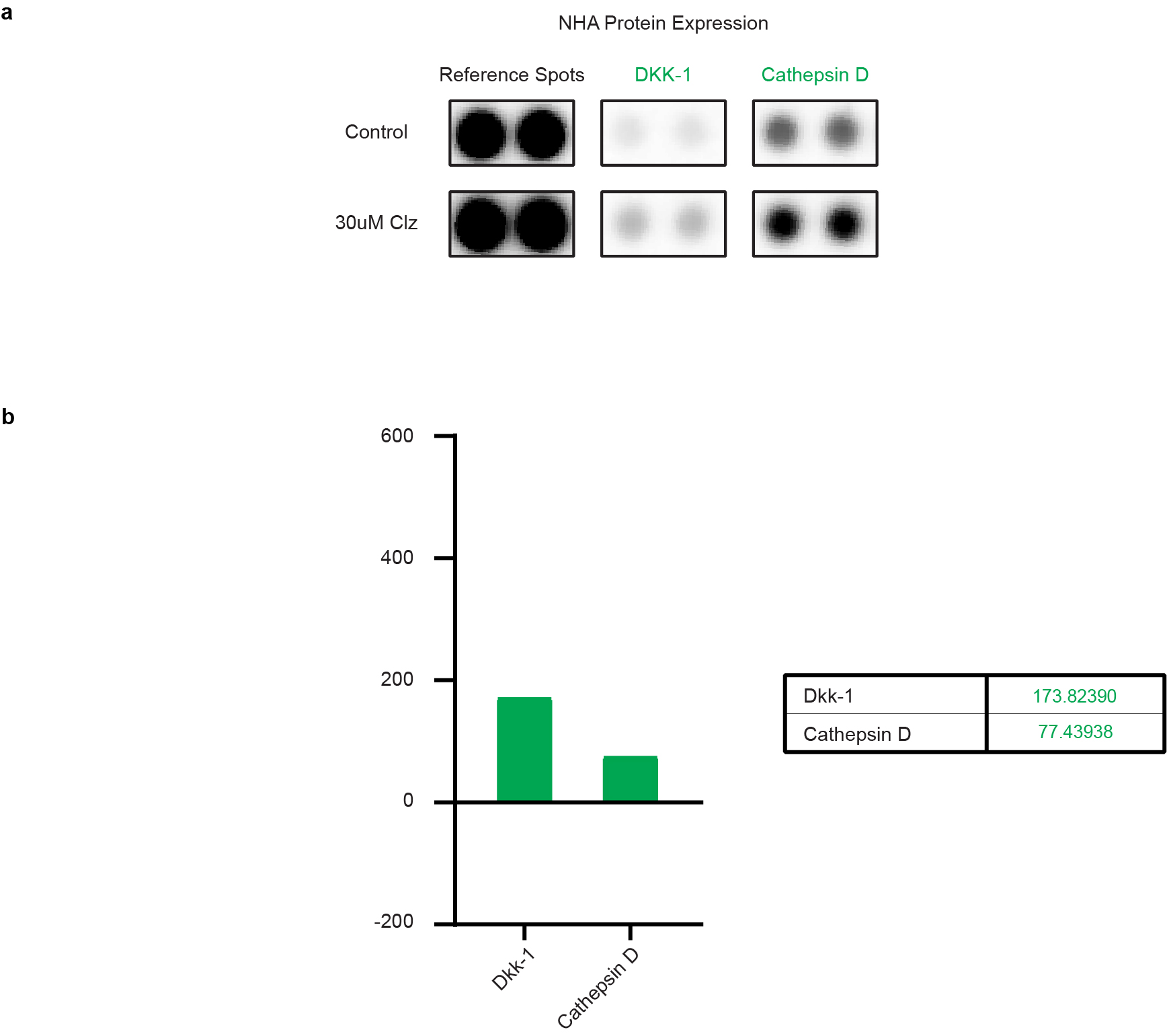

Supplement: Supplementary file 2 — Supplementary Material 2 [file 10585_2025_10328_MOESM2_ESM.jpg]

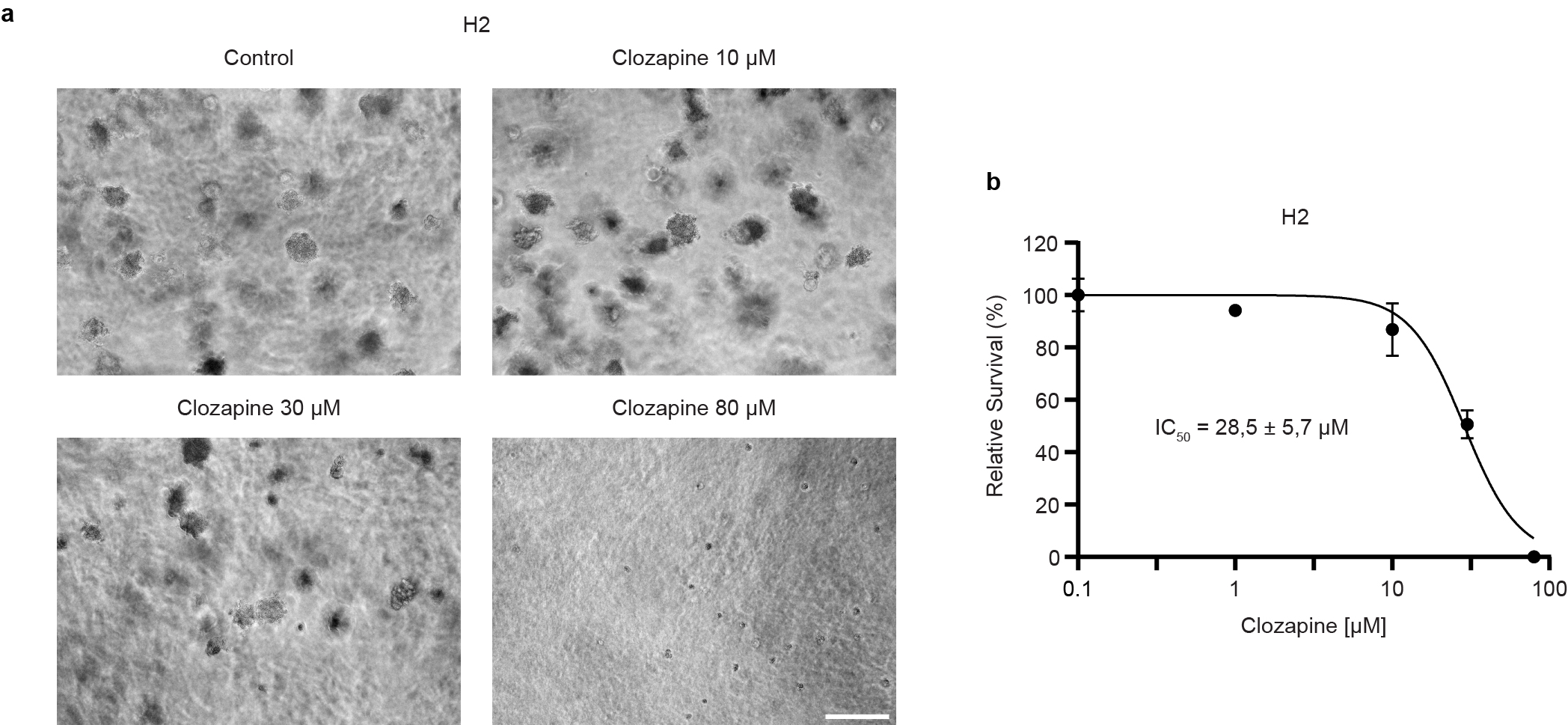

Supplement: Supplementary file 3 — Supplementary Material 3 [file 10585_2025_10328_MOESM3_ESM.jpg]

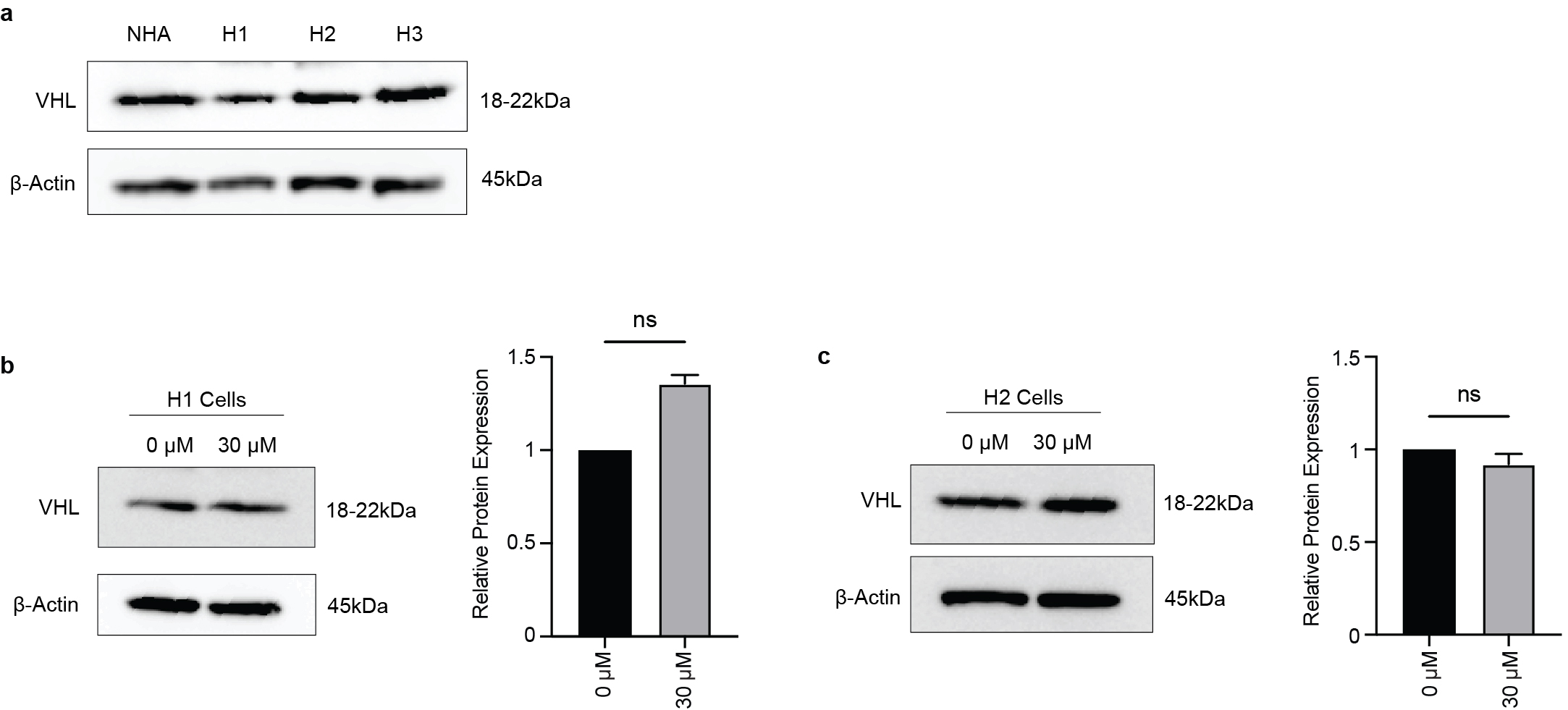

Supplement: Supplementary file 4 — Supplementary Material 4 [file 10585_2025_10328_MOESM4_ESM.jpg]

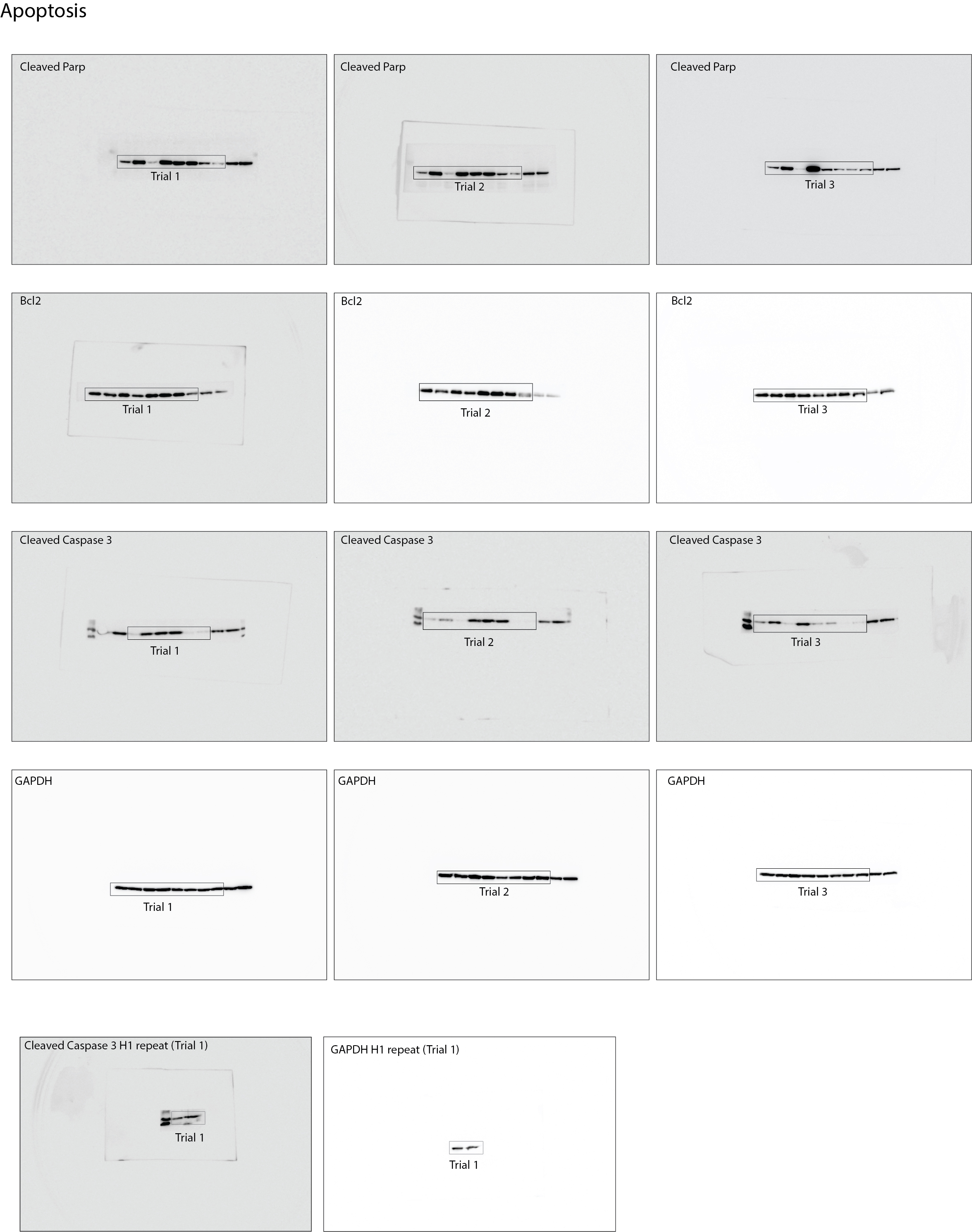

Supplement: Supplementary file 5 — Supplementary Material 5 [file 10585_2025_10328_MOESM5_ESM.jpg]

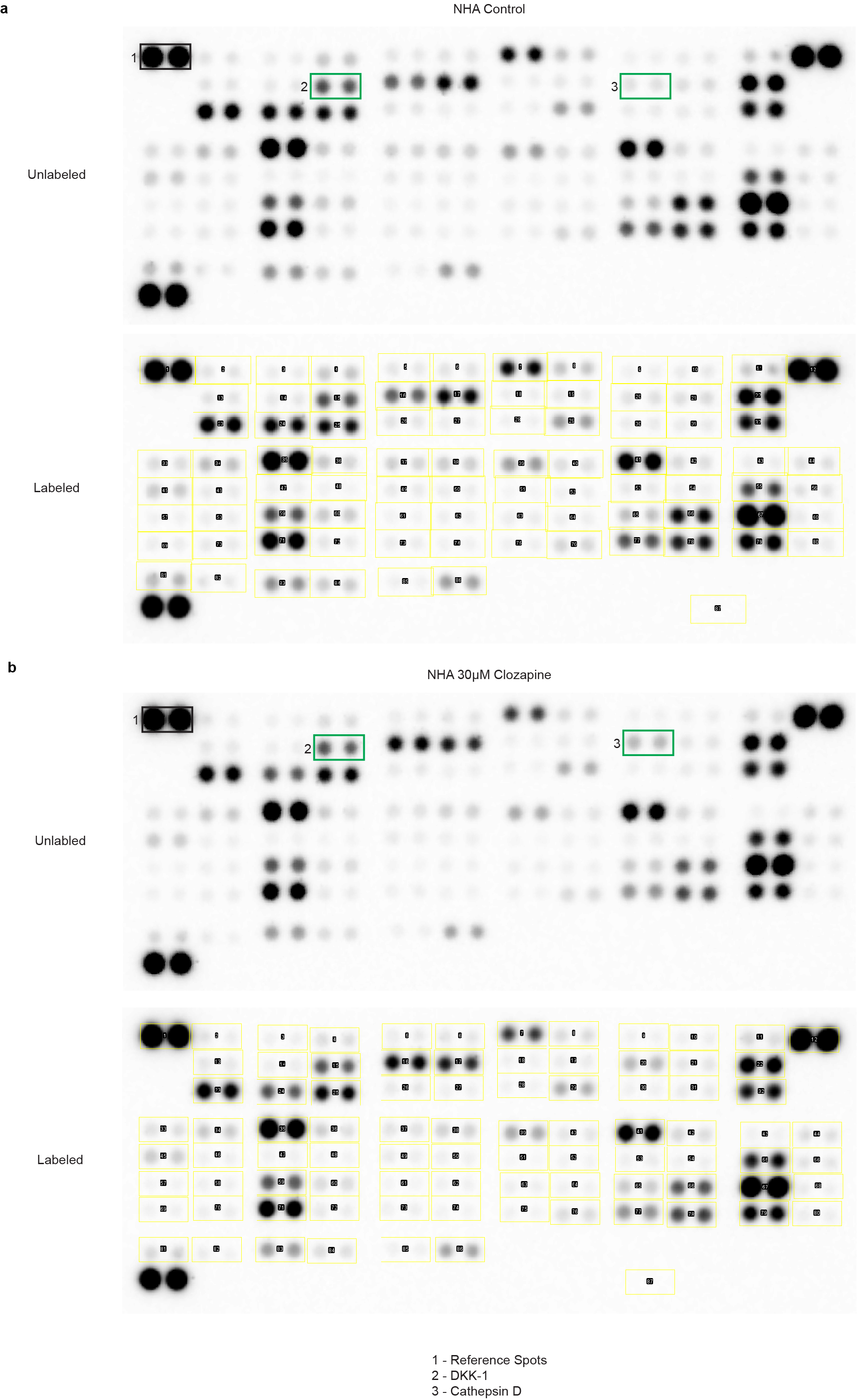

Supplement: Supplementary file 6 — Supplementary Material 6 [file 10585_2025_10328_MOESM6_ESM.jpg]

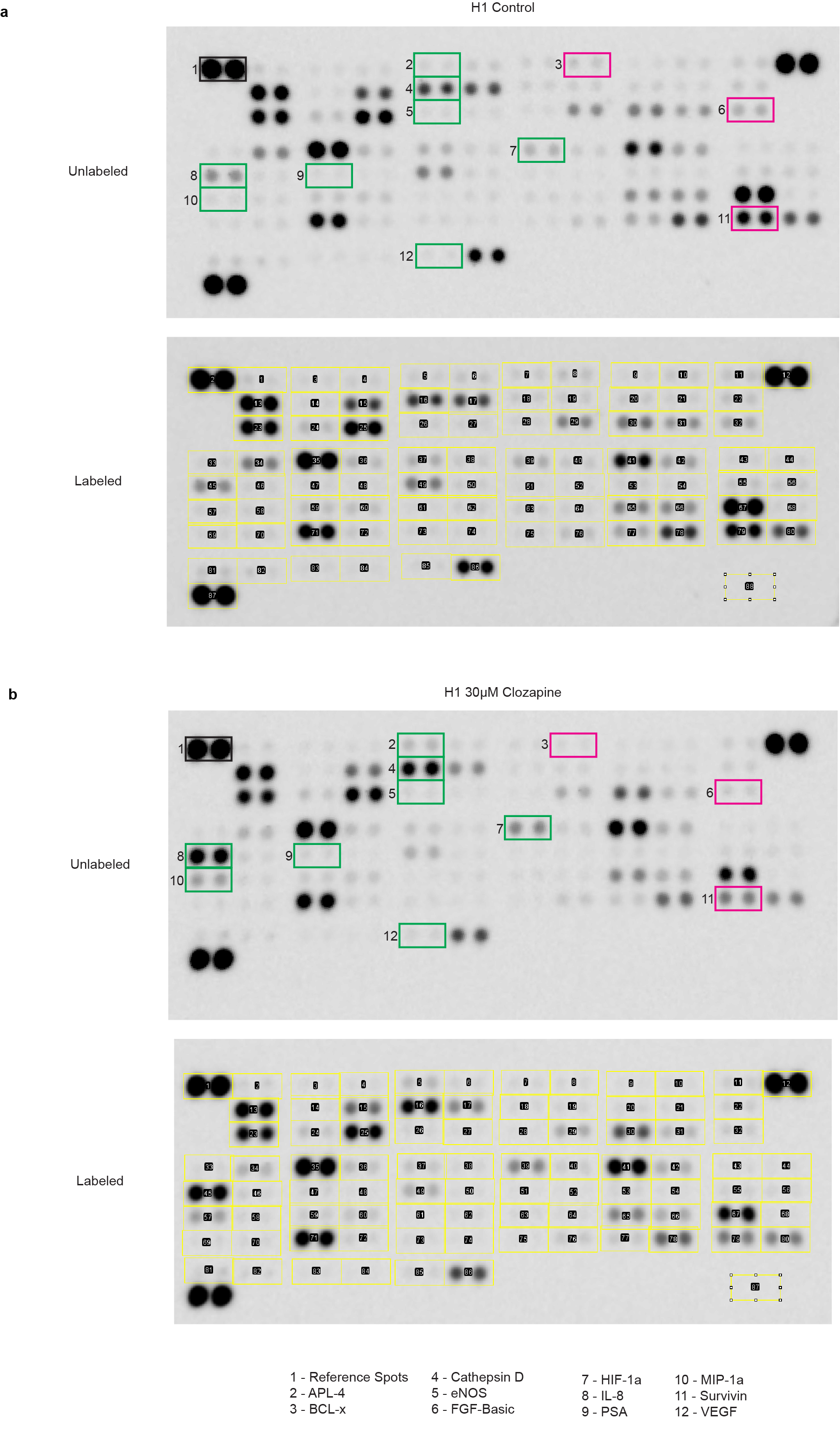

Supplement: Supplementary file 7 — Supplementary Material 7 [file 10585_2025_10328_MOESM7_ESM.jpg]

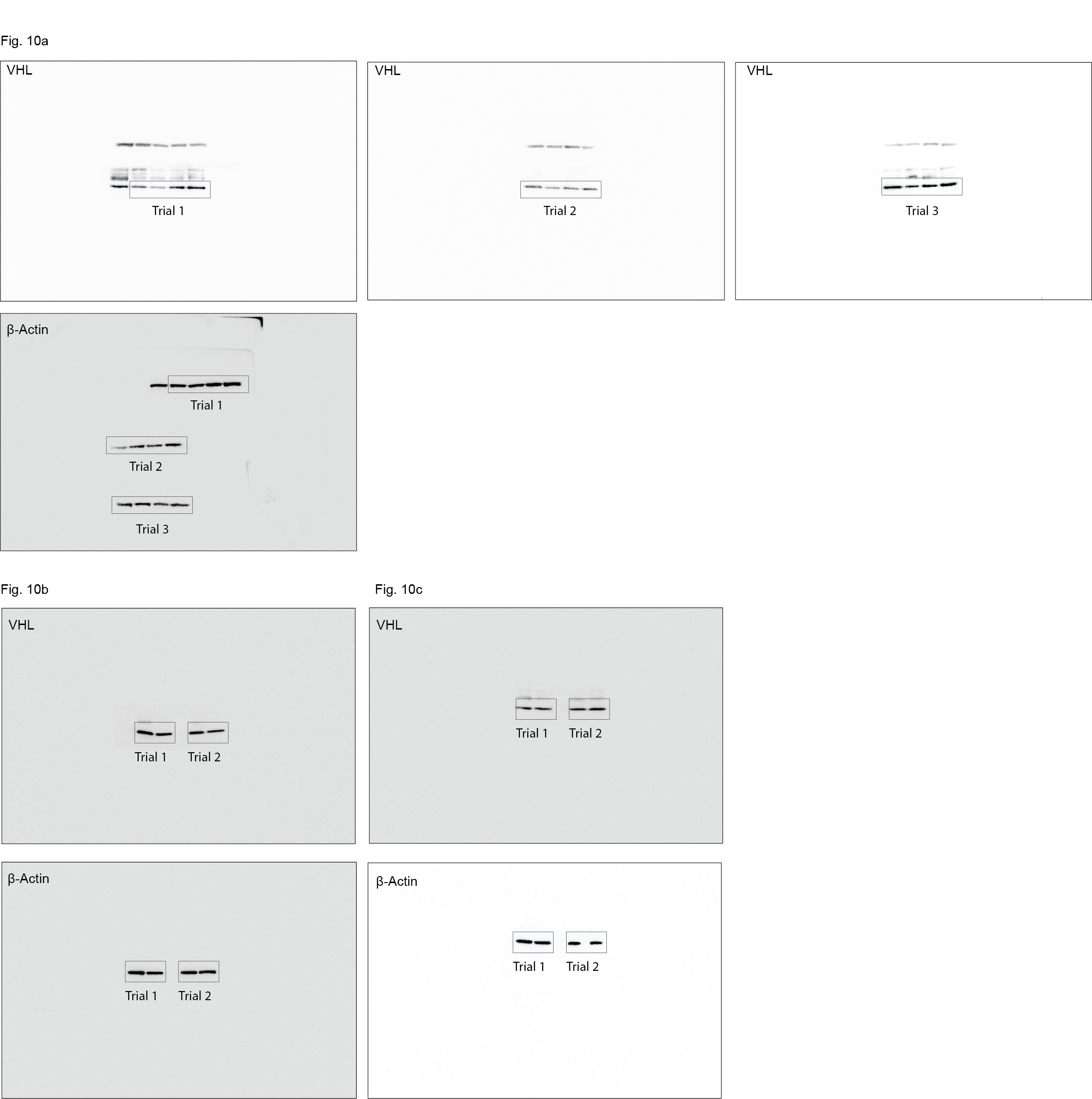

Supplement: Supplementary file 8 — Supplementary Material 8 [file 10585_2025_10328_MOESM8_ESM.jpg]

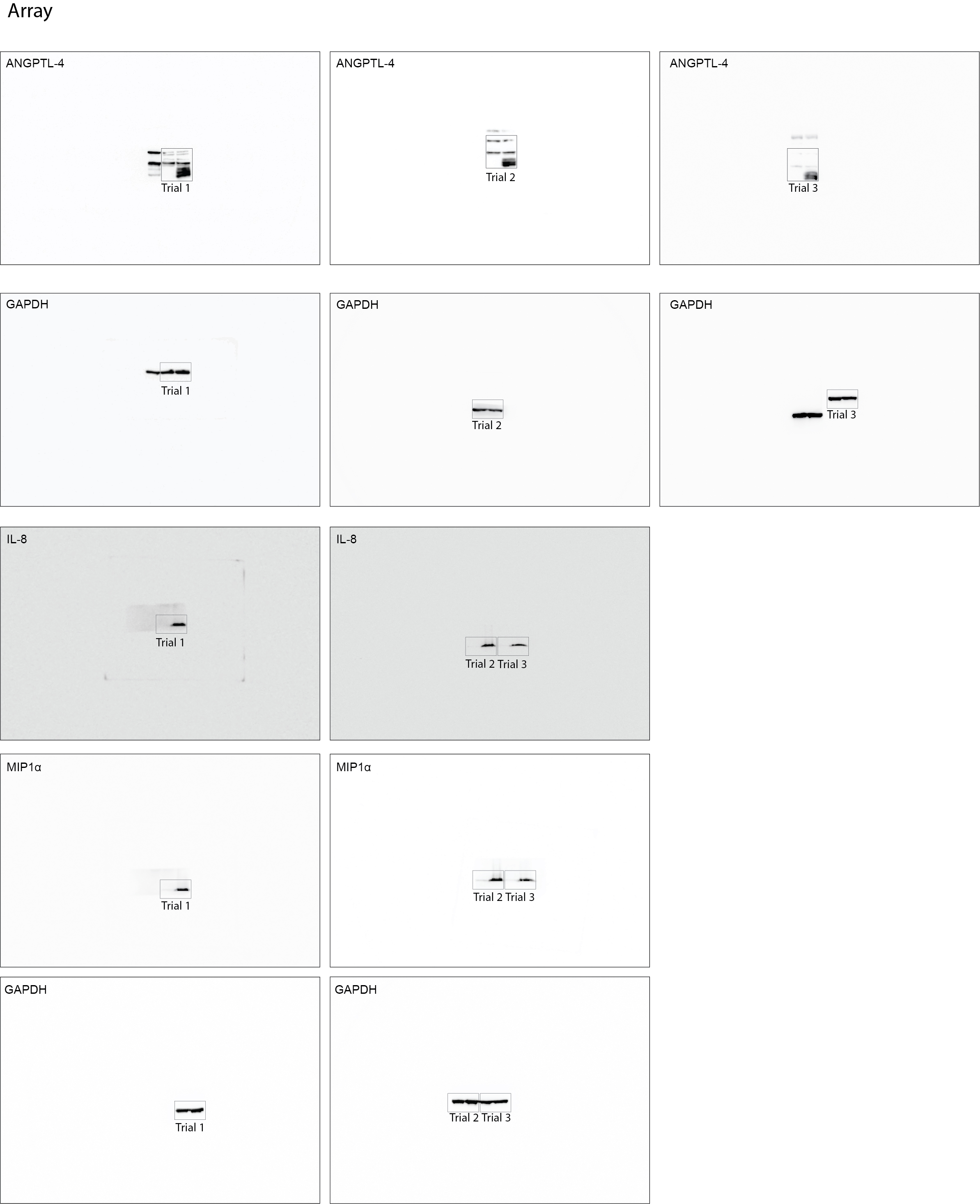

Supplement: Supplementary file 9 — Supplementary Material 9 [file 10585_2025_10328_MOESM9_ESM.jpg]

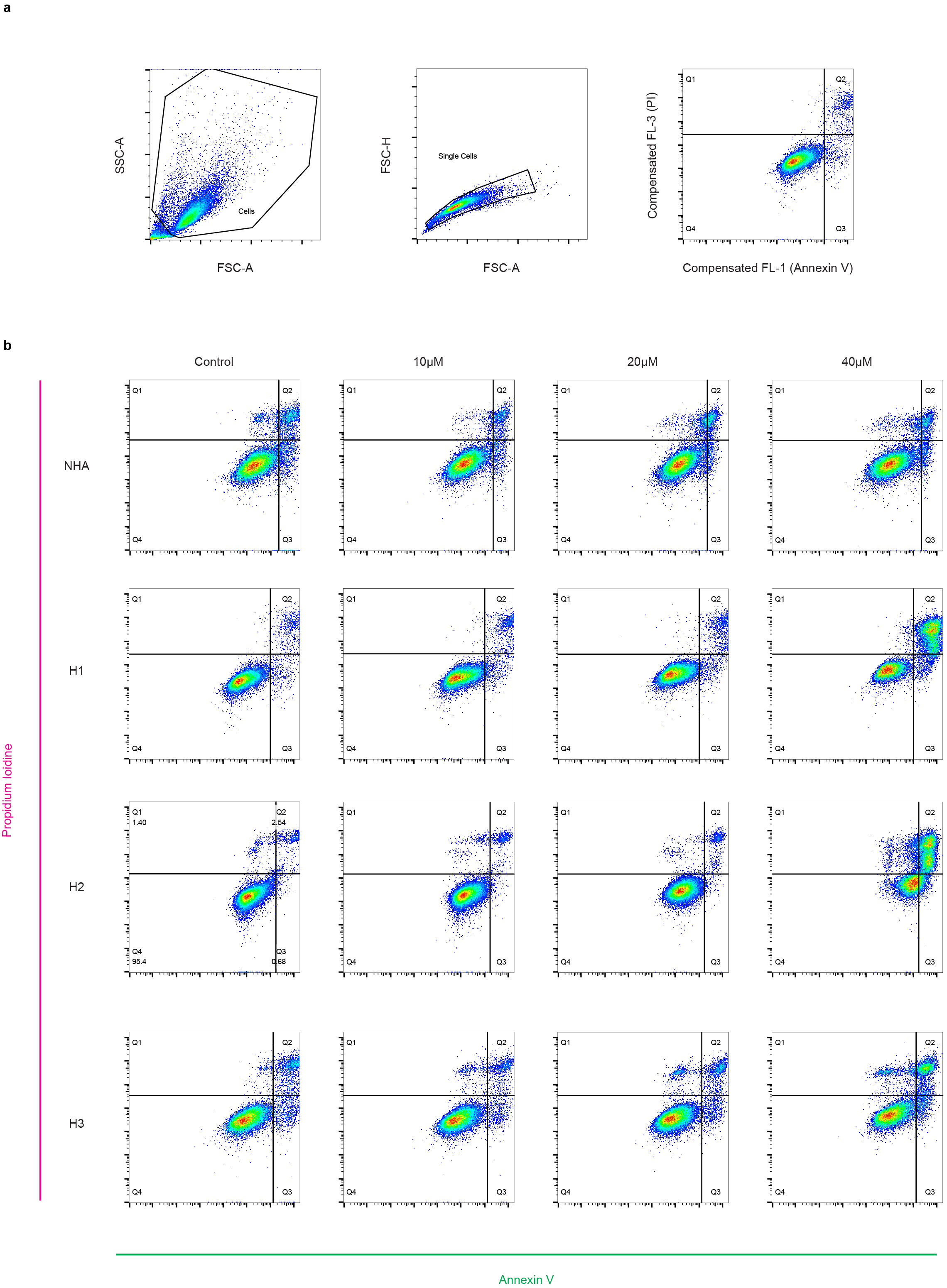

Supplement: Supplementary file 10 — Supplementary Material 10 [file 10585_2025_10328_MOESM10_ESM.jpg]

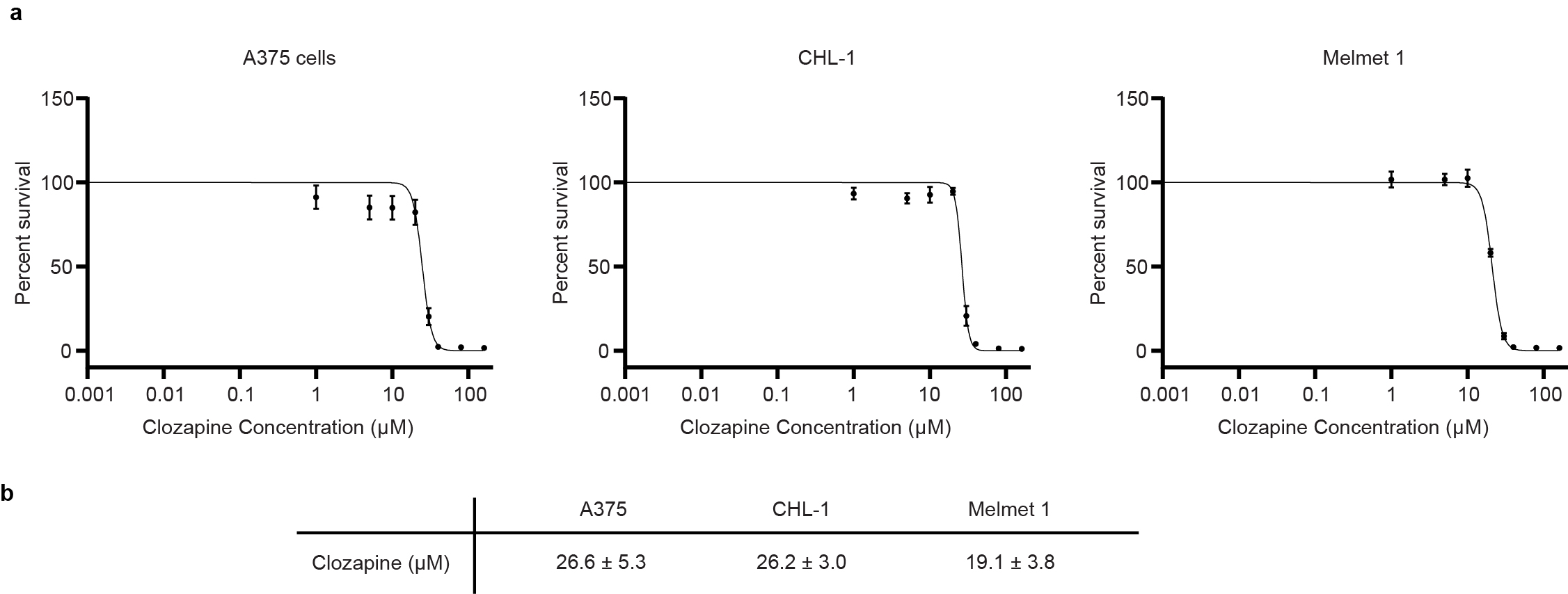

Supplement: Supplementary file 12 — Supplementary Material 12 [file 10585_2025_10328_MOESM12_ESM.jpg]

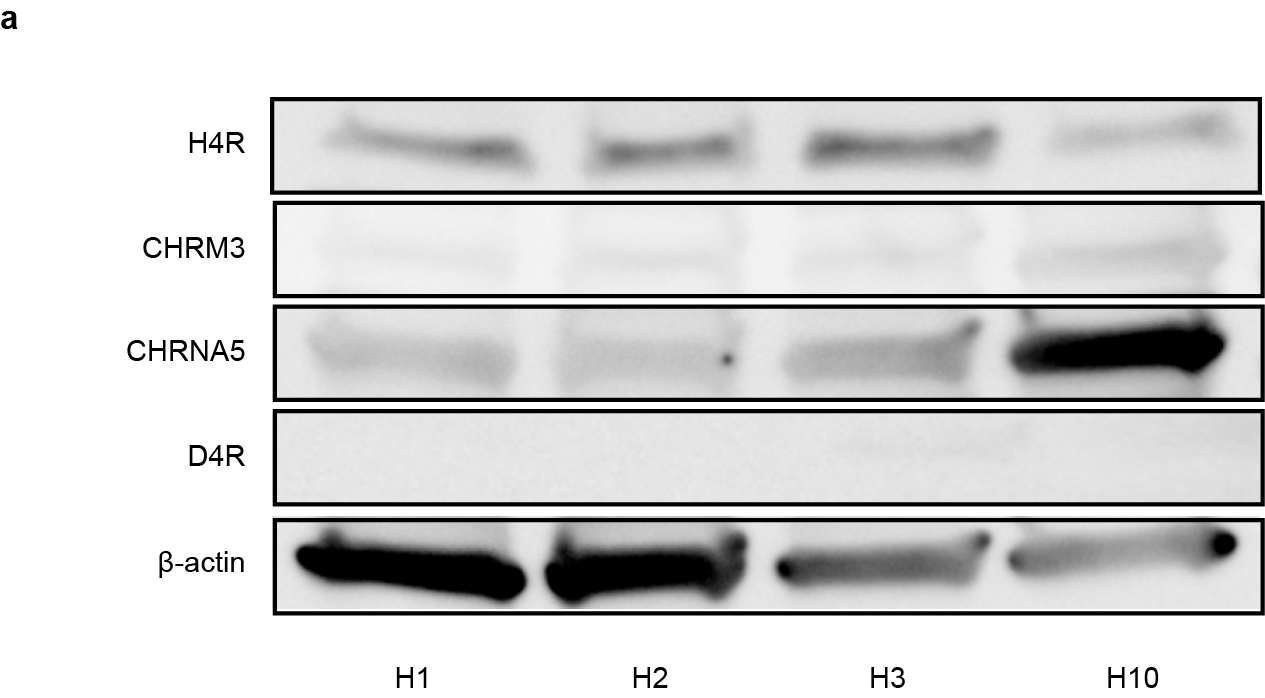

Supplement: Supplementary file 13 — Supplementary Material 13 [file 10585_2025_10328_MOESM13_ESM.jpg]

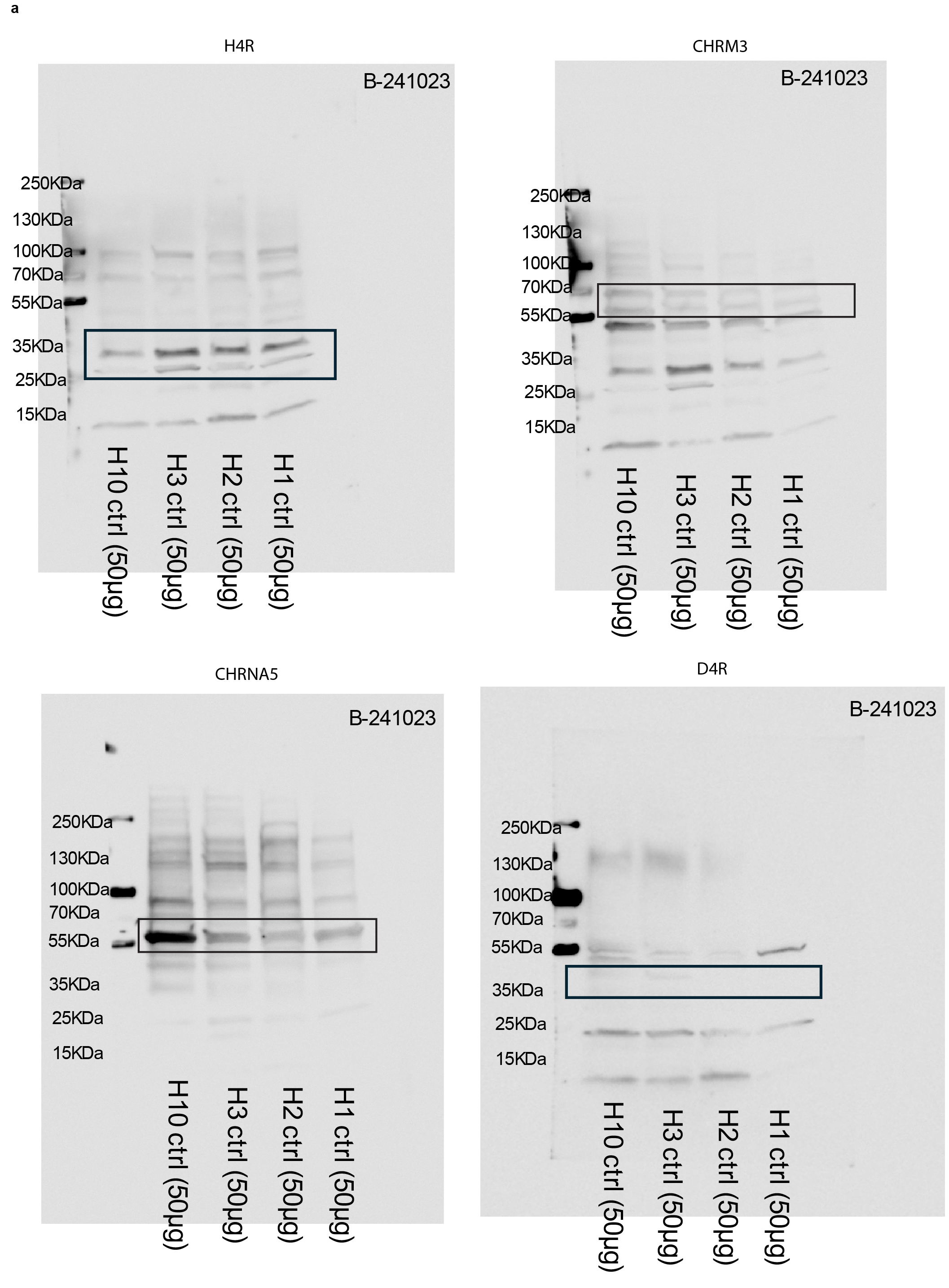

Supplement: Supplementary file 14 — Supplementary Material 14 [file 10585_2025_10328_MOESM14_ESM.jpg]
