# Supplementary material for: Repurposing neuroleptics: clozapine as a novel, adjuvant therapy for melanoma brain metastases
Source: Clin Exp Metastasis. 2025 Jan 25;42(2):12. doi: 10.1007/s10585-025-10328-3 (PMC11761981; doi:10.1007/s10585-025-10328-3)
Supplement: Supplementary file 15 — Supplementary Material 15 [file 10585_2025_10328_MOESM15_ESM.docx]

**Online Resources**


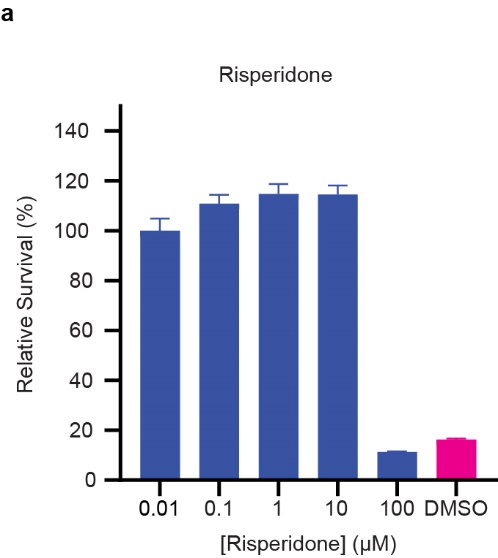


**Online Resource 1** Risperidone cytotoxicity is due to high DMSO concentrations. Graphical representation of risperidone cytotoxicity with drug solution at 0.1, 1 10 and 100 µM as compared to control. Risperidone has a low solubility in most solvents, in DMSO risperidone can exist at a maximum concentration of approximately 7mM. All other drugs from our screening were easily dissolved into DMSO at concentrations of 100 mM as stock solutions. As a result, drugging with 100 µM of risperidone results in a significant amount of DMSO added to the wells. The purple bar shows the same amount of DMSO added to the wells as when 100 µM was added to the wells.


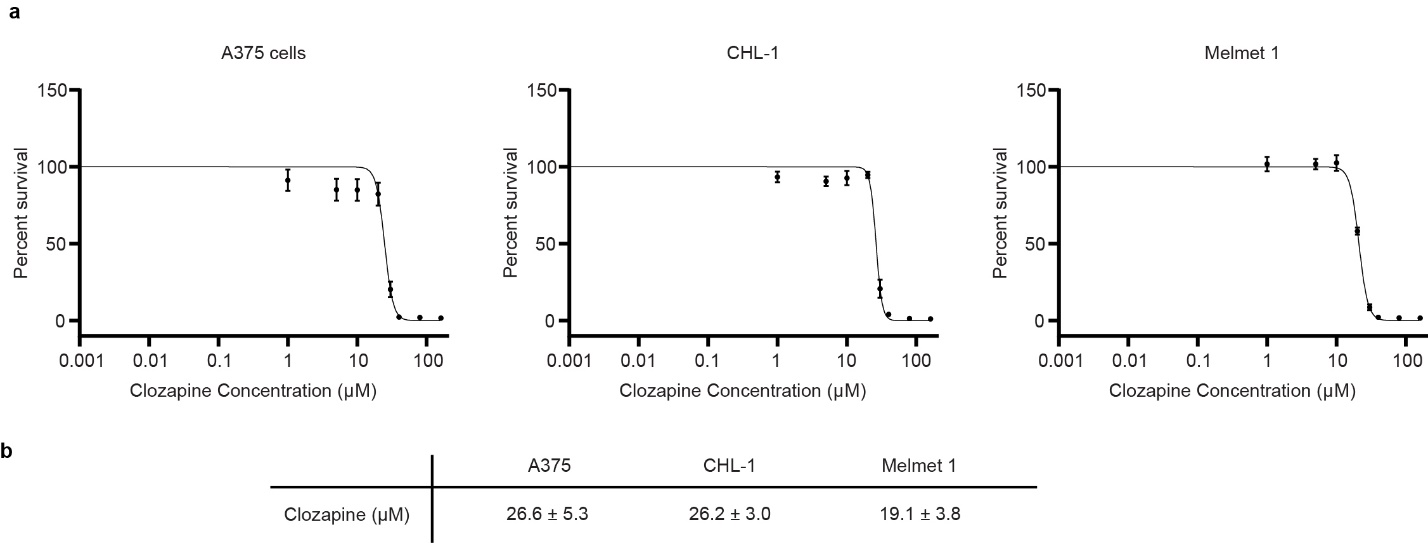


**Online Resource 2** Clozapine displays cytotoxic effects in primary melanoma cell lines. Graphical representation of clozapine cytotoxicity in primary melanoma cell lines A375 and CHL-1 as well as the lymph node metastatic cell lines Melmet-1. Cells were treated with clozapine at concentrations of 1, 5, 10, 20, 30, 40, 80, and 160 µM. Cells left in untreated medium were included as a baseline point.


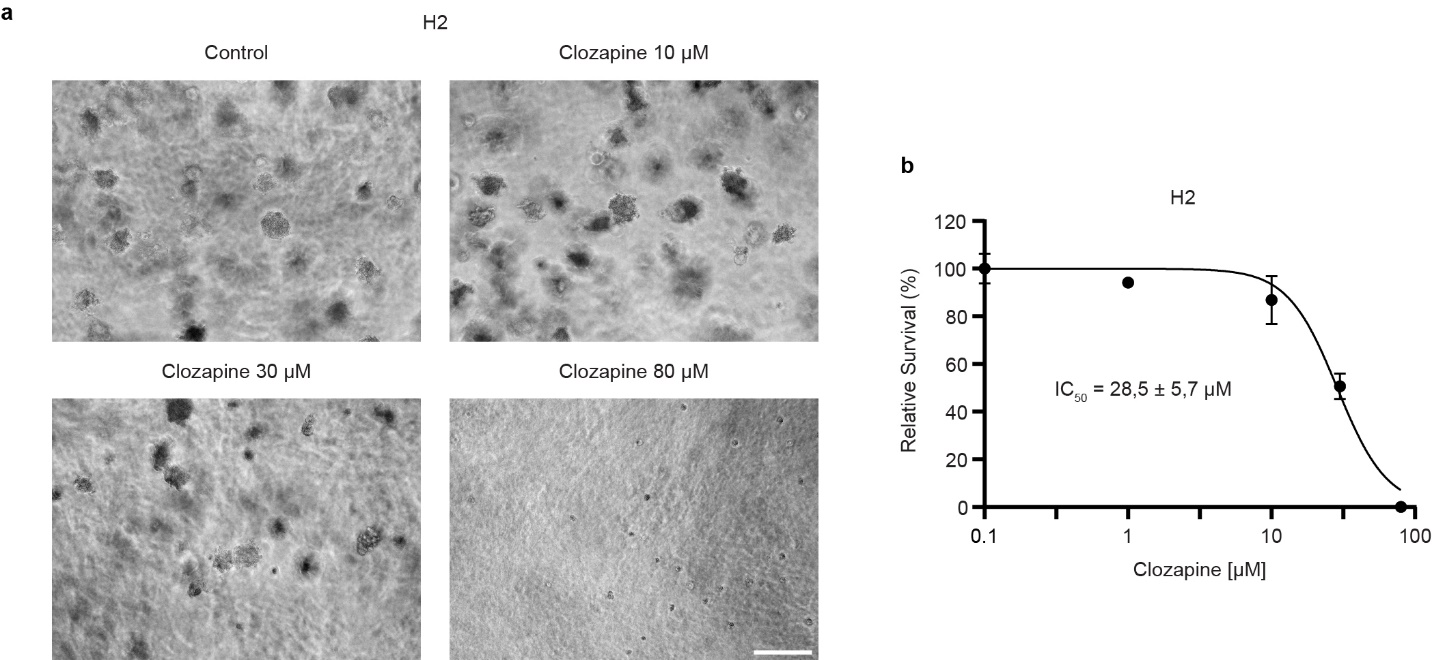


**Online Resource 3** Effective inhibition of tumor growth in a 3D tumorsphere assay. (a) Light microscopy images showing H2 MBM cell colonies suspended in soft agar after 14 days of clozapine treatment as compared to untreated controls. Scale bar = 200 µm. (b) Representative figure of IC_50_ doses of H2 cells in an anchorage independent model, graphically represented (n = 3).


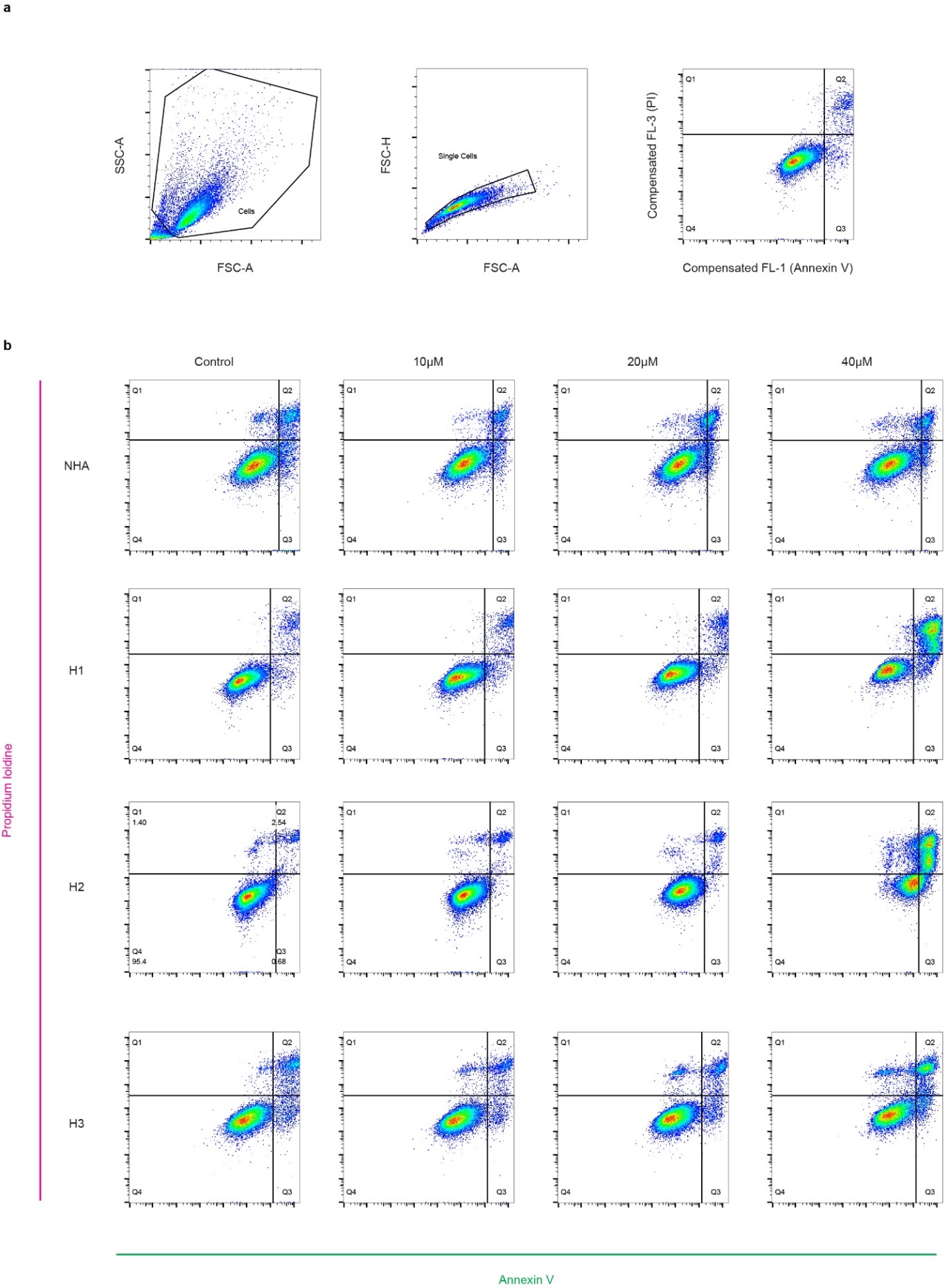


**Online Resource 4** Representation of data processing during flow analysis and a full overview of clozapine effects in different cell lines at different concentrations. (a) Gating and compensation. The left image shows gating to rule out debris. Middle shows gating to select single cells, and rule out clustered cells. Right shows an example of final gating to select quadrats representative of viable cells, early apoptosis, late apoptosis, and necrosis. Q4, Q3, Q2 and Q1, respectively. (b) Heat map representation of flow cytometry performed utilizing PI and Annexin-V to stain for apoptosis. Represented cell lines are normal human astrocytes (NHA), as well as our melanoma brain metastases cell lines H1, H2, and H3.


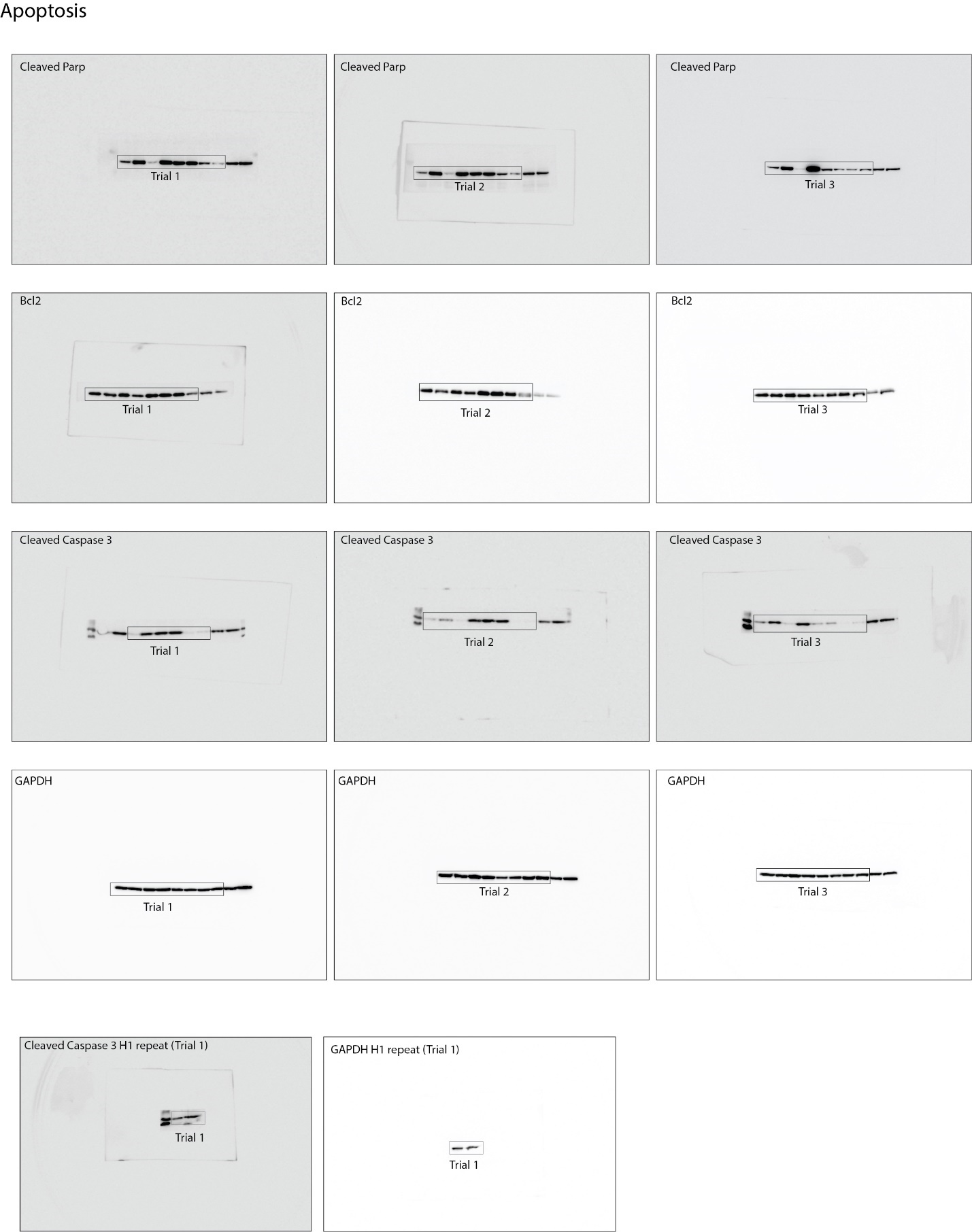


**Online Resource 5** Full blots of western blot analysis for validation of apoptosis. Stains for cleaved poly(ADP-ribose) polymerase (Cleaved PARP), B-cell leukemia/Lymphoma 2 (bcl-2), and cleaved caspase 3 (CC3). GAPDH was used as a control stain.


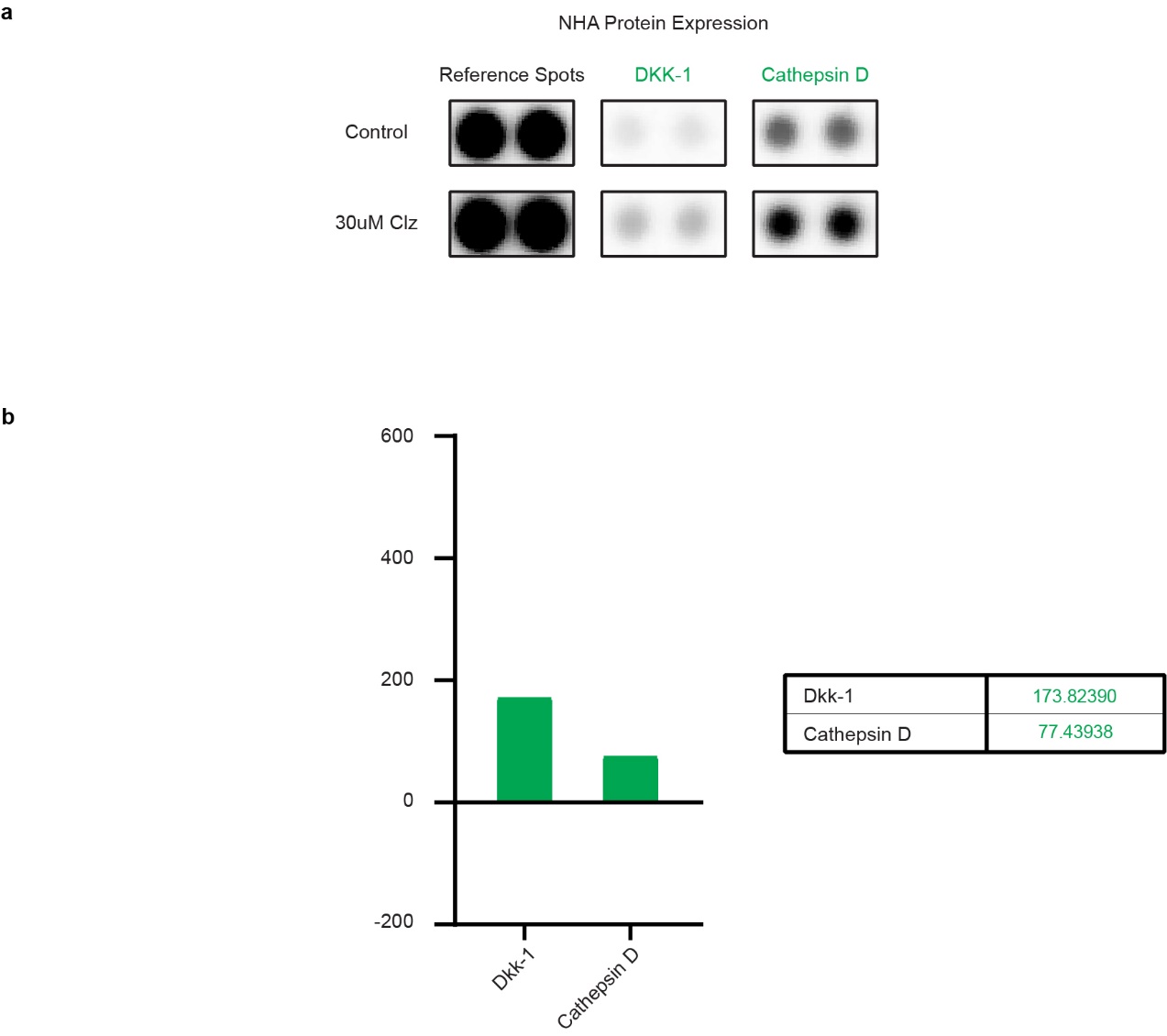


**Online Resource 6** Proteome profiler of NHA. (a) Raw blots from the protein profiler. Two blots per sample, comparing NHA cells as untreated controls to 72 h of clozapine treatment at 30 µM. (b) A graphical representation of the protein profiler results showing percentage differences in pixel density comparing NHA cells treated with clozapine for 72 h to untreated controls. Table showing proteins that were up regulated by more than 50%; in green. No proteins were downregulated by more than 50%.


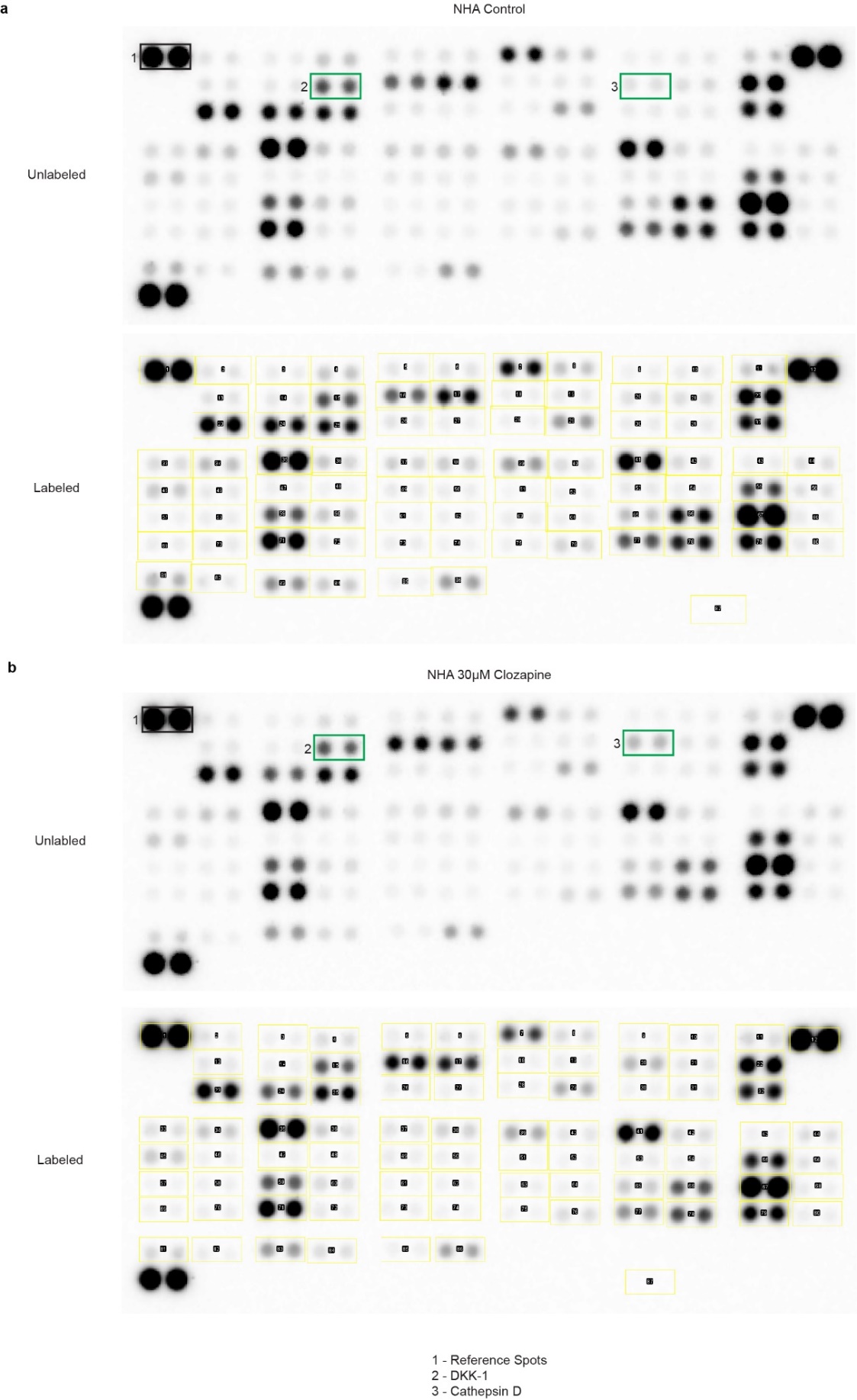


**Online Resource 7** Full blots from the protein profiler of NHA showing areas selected for pixel density analysis and regions that were found to differ by more than 50% between control and 72h of 30uM clozapine treatment. (a) Untreated controls. The top blot shows pre-analysis blot with regions of upregulated proteins marked with green rectangles, and control blots marked with a black rectangle. The number next to the marking represents the protein targeted and can be referenced at the bottom of the figure. The bottom blot shows regions selected for pixel density analysis which was analyzed and presented in supplementary figure 4 (b) Cells treated with 30uM clozapine for 72 hours. Blots follow the same layout as untreated controls.


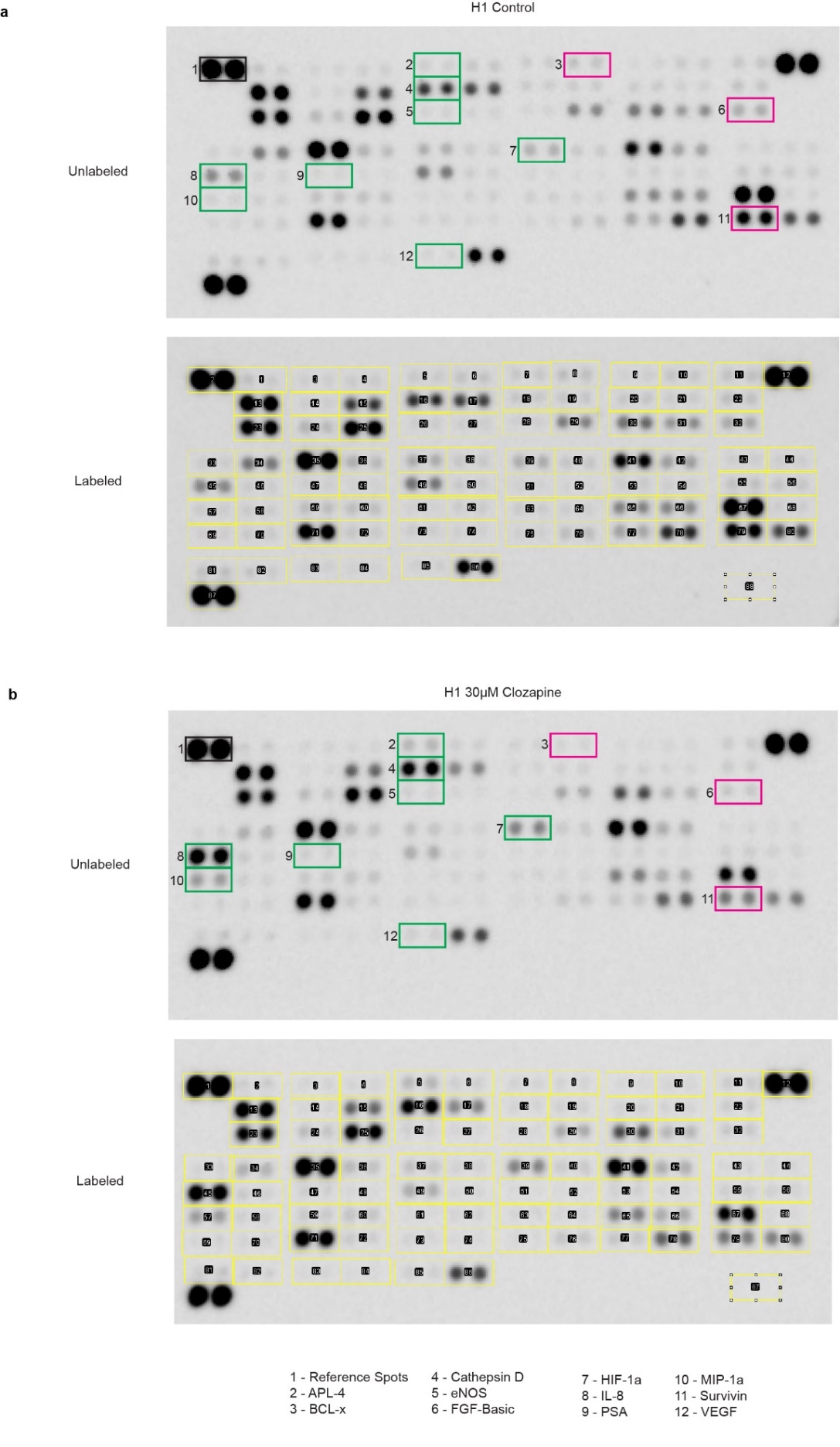


**Online Resource 8** Full blots from the protein profiler of H1 showing areas selected for pixel density analysis and regions that were found to differ by more than 50% between control and 72h of 30uM clozapine treatment. (a) Untreated controls. The top blot shows pre-analysis blot with regions of upregulated proteins marked with green rectangles, downregulated as magenta, and control blots marked with a black rectangle. The number next to the marking represents the protein targeted and can be referenced at the bottom of the figure. The bottom blot shows regions selected for pixel density analysis which was analyzed and presented in supplementary figure 4 (b) Cells treated with 30uM clozapine for 72 hours. Blots follow the same layout as untreated controls.


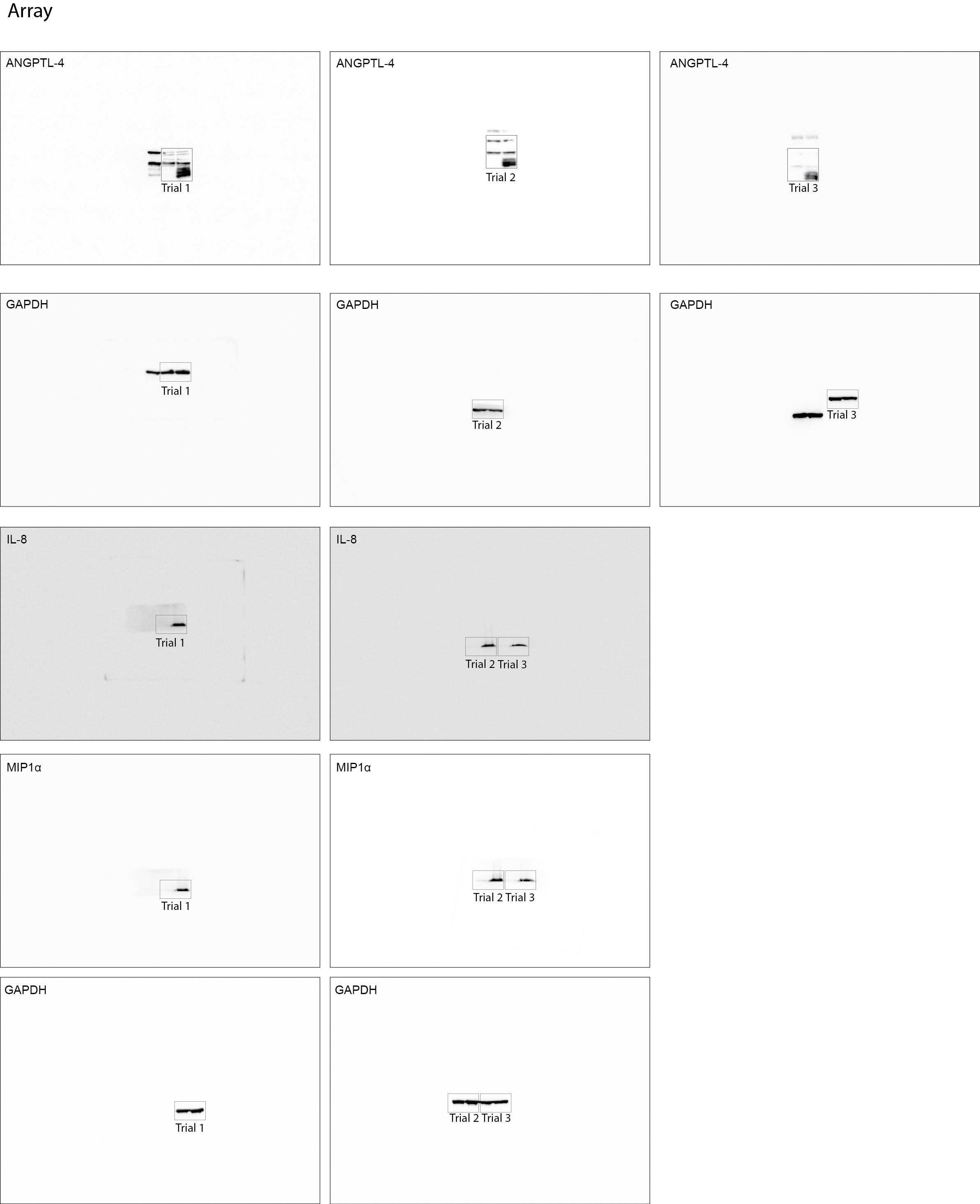


**Online Resource 9** Full blots of analysis seeking to validate results from the proteome profiler. Stains for interleukin 8 (IL-8), macrophage inflammatory protein 1 alpha (MIP1a), and angiopoietin-like 4 (ANGPTL-4). GAPDH was used as a control stain.


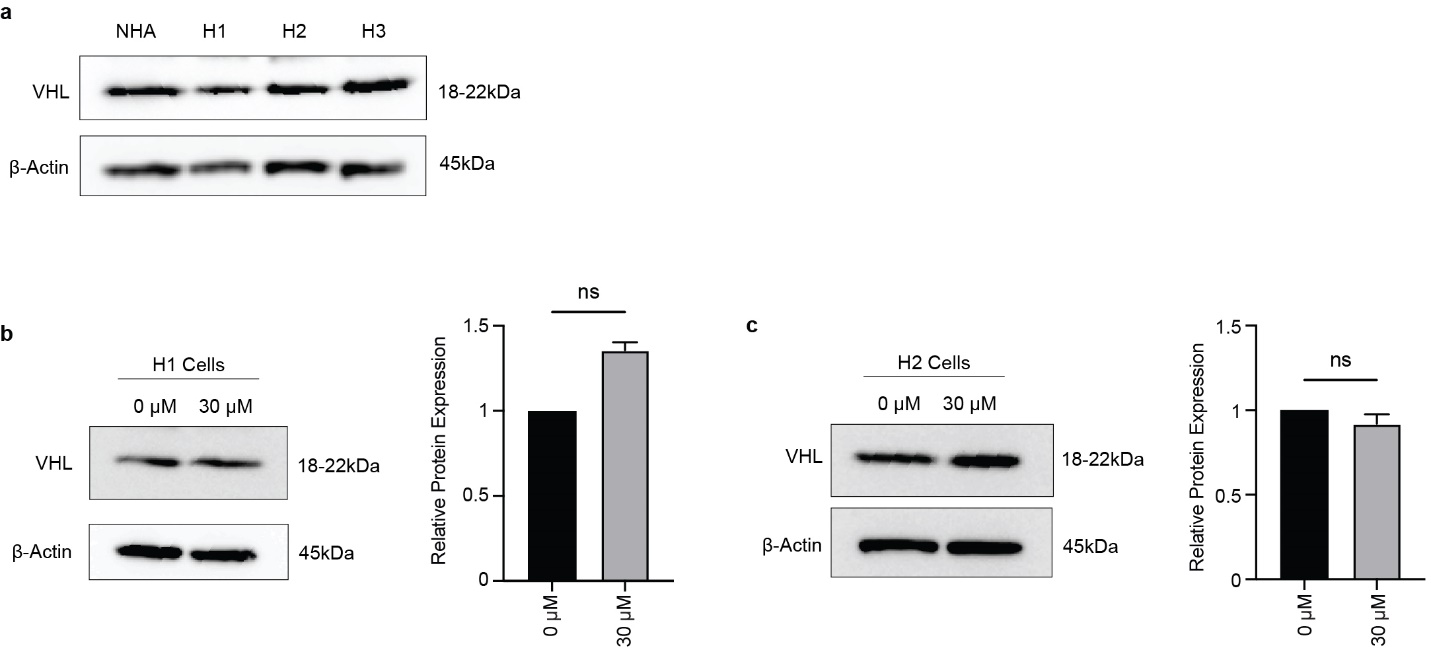


**Online Resource 10** Clozapine treatment does not induce changes in Von Hippel-Lindau (VHL) expression. (a) Western blots showing the presence of VHL in untreated cell lines normal human astrocytes (NHA) and the melanoma brain metastases cell lines H1, H2, and H3. β-actin is used as a control stain (b) Blots showing expression of VHL in H1 cells treated with 30uM clozapine for 72 h as compared to negative controls. β-actin was used as a control stain. Quantification of blot density showing no significant VHL up- or downregulation of VHL expression. (n=2) (c) Blots showing expression of VHL in H2 cells treated with 30uM clozapine for 72 h as compared to negative controls. β-actin was used as a control stain. Quantification of blot density showing no significant VHL up- or downregulation of VHL expression (n=2). ns = p > 0.05


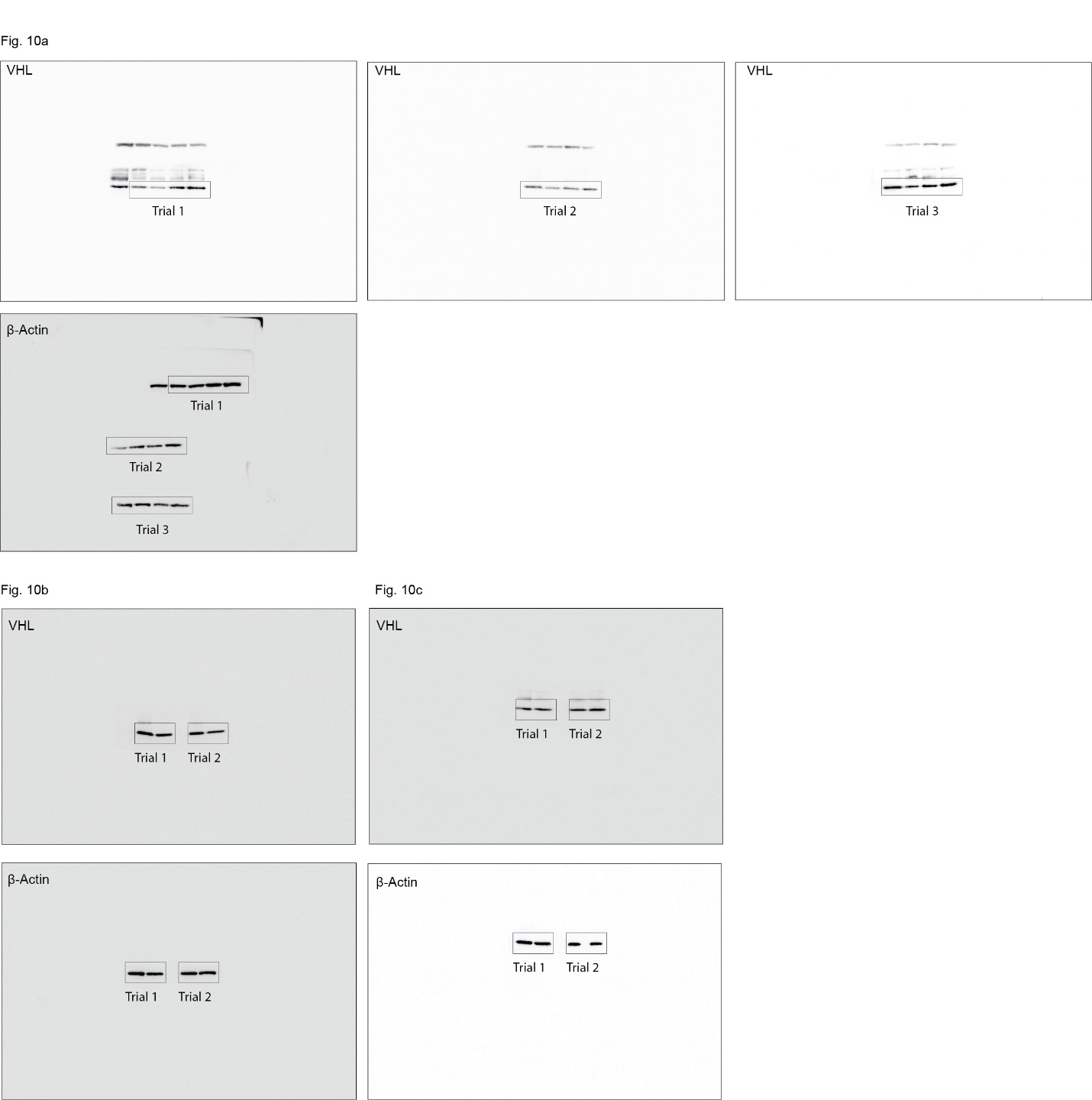


**Online Resource 11** Full blots of western blot analysis for expression of Von Hippel-Lindau (VHL) following clozapine treatment. Stains VHL, β-actin was used as a control stain.


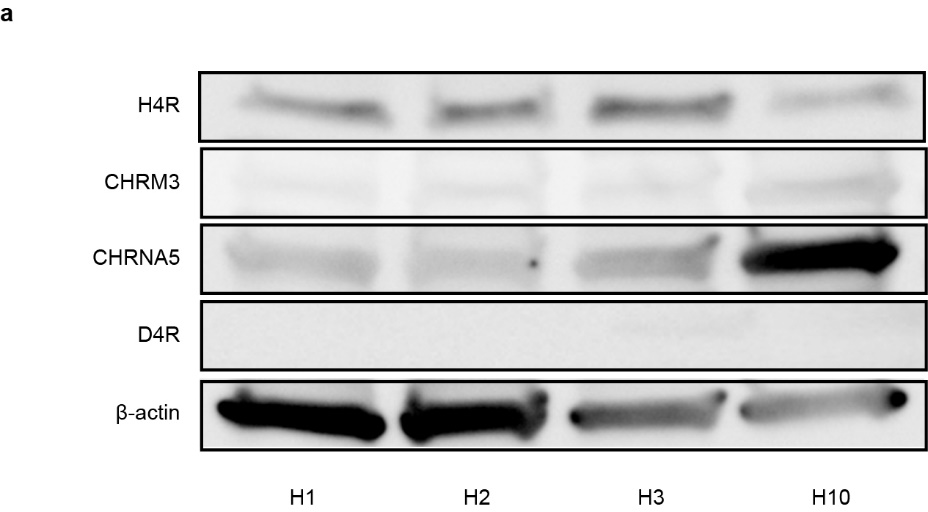


**Online Resource 12** Melanoma brain metastases cell cultures express histamine 4 receptor (H4R), muscarinic acetylcholine receptor 3 (CHRM3) and nicotinic acetylcholine receptor 5 (CHRNA5), but not the dopamine 4 receptor (D4R). (a) Western blots showing the presence of H4R. CHRM3 and CHRNA5 in untreated melanoma brain metastases cell lines H1, H2, H3 and H10. D4R expression is not discernable. β-actin is used as a control stain (n=3)


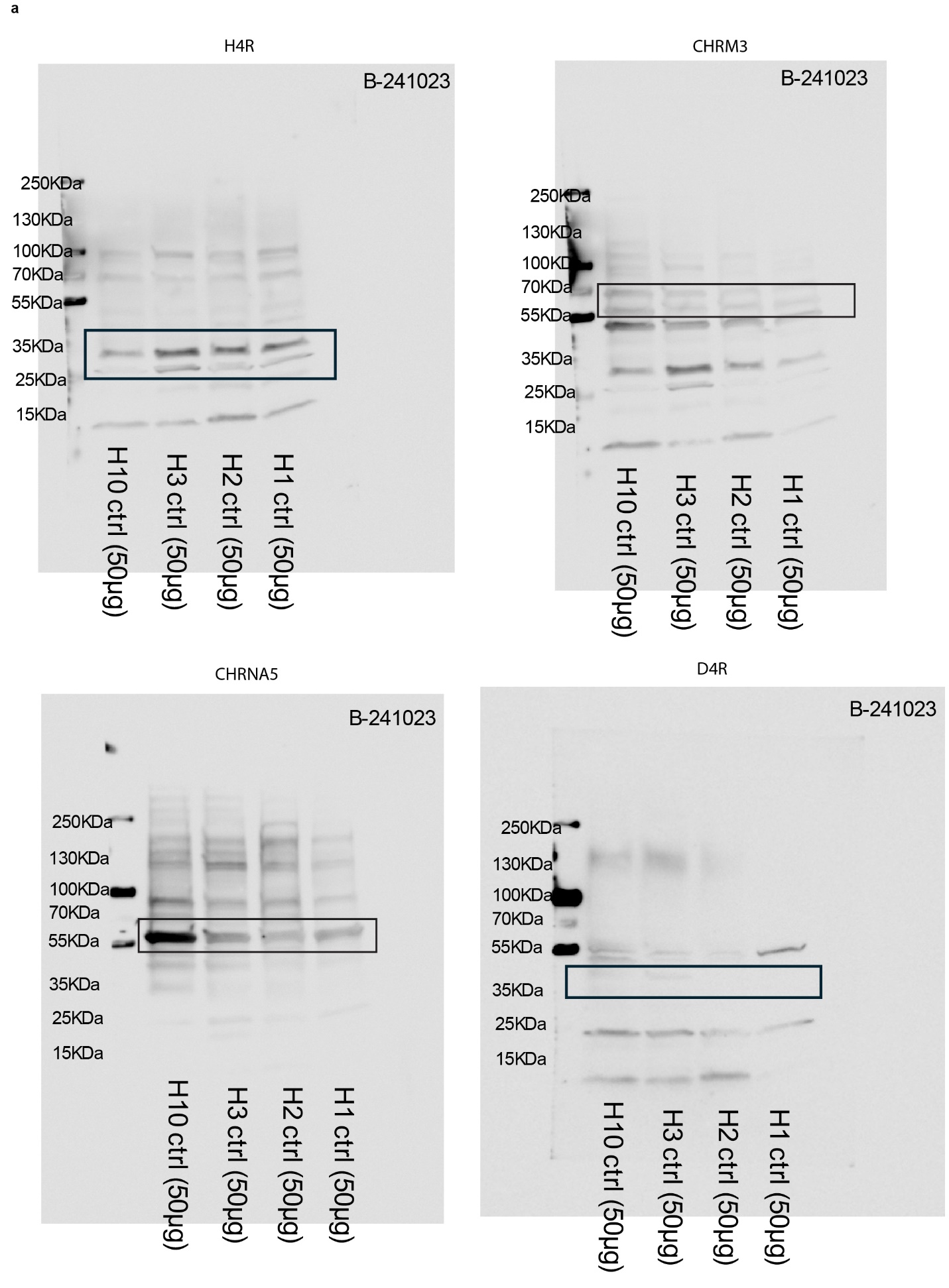


**Online Resource 13** Full blots of western blot analysis for expression of surface receptors related to clozapine in our melanoma brain metastases (MBM) cell lines. Stains for dopamine 4 receptor (D4R), histamine 4 receptor (H4R), muscarinic acetylcholine receptor 3 (CHRM3) and nicotinic acetylcholine receptor 5 (CHRNA5). β-actin was used as a control stain.
